# Cyclophilin J Reprograms Tumor-associated Macrophages to Exert an Anti-tumor Effect in Liver Cancer

**DOI:** 10.7150/ijbs.113197

**Published:** 2025-05-31

**Authors:** Jing Wang, Chen Yao, Qi Zeng, Lixia Peng, Shimeng Zhang, Yizhi Mao, Lingyi Fu, Shuai Chen, Chunjie Sheng

**Affiliations:** State Key Laboratory of Oncology in South China, Guangdong Provincial Clinical Research Center for Cancer, Sun Yat-sen University Cancer Center, Guangzhou 510060, People's Republic of China.

**Keywords:** Liver hepatocellular carcinoma (LIHC), Tumor-associated macrophages (TAMs), Cyclophilin J (CYPJ)

## Abstract

The presence of tumor-associated macrophages (TAMs) characterized by an M2-like phenotype sustains a robust immunosuppressive tumor microenvironment (TME), promoting liver hepatocellular carcinoma (LIHC) progression. Here, we find that genetic deletion of cyclophilin J (CYPJ) in mice significantly accelerates the development of liver cancer. Analysis of immune cell infiltration reveals that high expression of CYPJ correlates with an increased proportion of M1-polarized, anti-tumor macrophages and CD8^+^ T cells in the TME. Mechanistically, we demonstrate that CYPJ interacts with AKT1 and inhibits the PI3K-AKT signaling pathway, which leads to polarization of TAMs toward the anti-tumor M1 phenotype, resulting in a tumor-suppressive effect. Collectively, our findings implicate CYPJ as a novel potential therapeutic target for macrophage-mediated therapy in liver cancer.

## Introduction

Tumor-associated macrophages (TAMs) are among the most abundantly infiltrated immune cells in tumors, influencing tumor proliferation, invasion, metastasis, extracellular matrix remodeling, immune suppression, and chemoresistance 1, 2. In tumors, TAMs can be divided into classically activated type 1 macrophages (M1) and type 2 macrophages (M2) 3. M1 macrophages are activated by interferon-γ (IFN-γ), tumor necrosis factor (TNF) and lipopolysaccharide (LPS), among others, and exhibit significant tumor-killing and phagocytic effects 4, 5, while M2 TAMs can be stimulated by transforming growth factor (TGF), interleukin 4 (IL4) and macrophage colony-stimulating factor (M-CSF) et al., and often promote tumor cell growth and exhibit an immunosuppressive phenotype 6, 7. Given that macrophages possess the ability to activate the immune system and exhibit immune-killing functions, their application of macrophages to anti-tumor therapy is one of the most promising cancer treatment methods 8, 9. Therapeutic approaches involving macrophages encompass exhausting TAMs, enhancing their phagocytosis, and reprogramming TAMs 10, 11. Notably, among these, reprogramming TAMs from an M2-like phenotype to an M1-like phenotype offers the opportunity to maximize tumor killing 12, 13. M1 TAMs can secrete a variety of cytokines and produce inducible nitric oxide synthase (iNOS) to activate anti-tumor signal pathways 14, 15, and also promote the recruitment of immune cells like CD8^+^ T cells to eliminate tumors 16.

Cyclophilins are a group of proteins with peptidyl-prolyl cis-trans isomerase (PPIase) activity and are involved in protein folding and function regulation 17. Cyclophilin J (CYPJ), also named peptidylprolyl isomerase like 3 (PPIL3), has been reported by us to participate in inflammation regulation by inhibiting the ubiquitin chain 18. Our recent study revealed that it suppresses colorectal cancer (CRC) development by competing with the ubiquitin chain 19. Given the roles of CYPJ in regulating inflammation and cancers, we further wanted to explore whether CYPJ also plays a role in tumor immune microenvironment, and so far, its role in TME remains unknown.

In the current study, we observe that CYPJ expression is induced in M1 macrophages and is associated with an increased M1 TAMs population in LIHC. Mechanistically, CYPJ binds to AKT1 and inhibits the AKT signal pathway, thus promoting TAMs to polarize toward the M1 phenotype. Overall, our findings demonstrate that CYPJ can reprogram macrophages to exert an anti-tumor effect, highlighting its potential as a new target of immune cell therapy for LIHC.

## Materials and Methods

### Mice

The *Cypj*-deficient mice were generated by CRISPR-Cas9 gene targeting technology (Shanghai Biomodel Organism Science & Technology Development Co. Ltd., Shanghai, China). C57BL/6 wild-type mice were purchased from Zhuhai BesTest Bio-Tech Co.,Ltd (Zhuhai, China). All mice were housed in a Specific Pathogen Free (SPF) grade environment. The Institutional Animal Care and Use Committee at Sun Yat-Sen University (SYSU, Guangzhou, China) approved all animal experiments in this study (Approval number: SYSU-IACUC-2024-002751).

### Animal Experiments

#### Hydrodynamic transfection experiments

A total of 2-2.5 mL of isotonic physiological solution containing the AKT, NRAS^G12V^ (a persistently active form of NRAS, called NRAS for short), and SB (a sleeping beauty transposon system) plasmids was injected through the tail vein in 5-7 seconds 24. The huge pressure primarily directs the plasmids to enter liver cells, where they are expressed. After 4-5 weeks, the male mice were sacrificed, and the formation of tumors could be directly observed on the surface of the liver.

#### Bone marrow chimeras (BMC)

One week before irradiation, fed the recipient male mice with neomycin water (5 g/L). Recipient mice were lethally irradiated with 9.5 Gy/20 g at 8-12 weeks. Six hours later, bone marrow cells (5×10^6^) harvested from the donor male mice were injected intravenously into irradiated recipients. Continue to feed the mice with neomycin water (5 g/L) for another two weeks. After two weeks, change back to regular drinking water and wait for the immune system reconstitution for six weeks.

#### Subcutaneous tumor model

The 1×10^6^ Hepa1-6 cells were injected subcutaneously into mice. And for the subcutaneous mixed tumor models in mice, Hepa1-6 cells mixed with mouse primary bone marrow-derived macrophages (BMDMs) at a ratio of 1:1, a total of 1 × 10^6^ cells. Tumor volume was measured every two or three days. The endpoint for measurement was reached when the tumor volume in any mouse exceeded 2000 mm^3^. After that, the tumors were removed for further analysis.

### Bioinformatics Analysis

The survival analysis of CYPJ was investigated utilizing the liver cancer data available on the Kaplan-Meier Plotter website (https://kmplot.com/analysis/) 20. The single-cell data were analyzed on TISCH1 (http://tisch1.comp-genomics.org/) 21. Subsequently, the online tool BSET (https://rookieutopia.com/app_direct/BEST/) was used to explore the correlation between CYPJ and the tumor-infiltrating immune cells in LIHC 22. The survival analysis regarding the expression of CYPJ and macrophages in LIHC was conducted on TIMER2.0 (http://timer.cistrome.org/) 23.

### RNA sequencing (RNA-seq)

Total RNA was extracted using the MagZol^TM^ Reagent kit (Magen, #R4801, China) according to the manufacturer's protocol. RNA quality was assessed on an Agilent 2100 Bioanalyzer (Agilent Technologies, USA). After that, eukaryotic mRNA was enriched and fragmented into short fragments. The sequencing service was performed by Annaroad Gene Technology (Beijing) Co., Ltd.

### Cell Culture

Hepa1-6, 293T, L929, Raji, K562 and RAW264.7 cells were stored in liquid nitrogen in the laboratory. Immortalized BMDM (iBMDM) cells were a kindly gift by Shao Feng (National Institute of Biological Sciences, Beijing). After cell resuscitation, the cells are cultured in DMEM (Gibco, USA) or RPMI 1640 medium with 10% fetal bovine serum (FBS, HyClone, USA) and added with 1% penicillin and streptomycin. All cells are cultured in an incubator at 37 ℃ with 5% CO_2_.

BMDMs were isolated from the C57BL/6 wild-type mice (WT-BMDM) and *Cypj*-KO mice (KO-BMDM). The isolated BMDMs were washed with PBS, centrifuged, and lysed to remove red blood cells, and then filtered through a 200-mesh sieve. Ultimately, BMDMs were resuspended in RPMI 1640 medium (Gibco, USA) containing 10% FBS, 1% penicillin mixed streptomycin, and 20 ng/mL of macrophage colony-stimulating factor (M-CSF, PeproTech, USA) or 20% L929 supernatant. The BMDMs were used after 5-7 days of differentiation. For macrophage polarization assays, BMDMs are stimulated with 100 ng/mL LPS (or 100 ng/mL LPS with 20 ng/mL IFN-γ, PeproTech, USA) for M1 polarization, and 20 ng/mL IL4 (PeproTech, USA) was used for M2 activation.

### Isolation of RNA and Quantitative Real-Time Polymerase Chain Reaction (qRT-PCR)

Total RNA was extracted using FreeZol Reagent (Vazyme, #R711, China) following the manufacturer's instructions. Afterward, the isolated RNAs were used for reverse transcription to synthesize cDNA (Vazyme, #R333, China). And the SYBR Green Master Mix (Genstar, #A301, China) was utilized for the qRT-PCR experiment. Finally, the Light Cycler 480 Real-Time PCR system (Roche, USA) was used to detect the expression of genes. The GAPDH serves as internal control. For the tissues of tumors, the 2^(-ΔCT)^ method was used for relative quantitation, while the relative quantitation of cells (like BMDMs) was performed with the 2^(-ΔΔCT)^ method. All the sequences of the primers are displayed in **Supplementary Table 1**.

### Flow Cytometry

To analyze infiltrating immune cells in tumors, tumor tissues were ground into single cells and washed with PBS, then filtered through a 200-mesh sieve and resuspended in a 5% BSA solution in preparation for staining. Next, the TAMs and T cells in the tumor were detected. The anti-tumor M1 TAMs were detected as CD45^+^/CD11b^+^/F4/80^+^/iNOS^+^ cells, whereas tumor-promoting M2 TAMs were CD45^+^/CD11b^+^/F4/80^+^/CD206^+^ cells. T cells were defined as CD4^+^ T cells and CD8^+^ T cells. All the surface markers were stained at 4 °C for 30 minutes in the dark, and the intracellular CD206 was stained after being permeabilized with the membrane-breaking reagent (BD Bioscience, #554722) according to the manufacturer's instructions. The relevant antibody information is recorded in **Supplementary Table 2**.

For the phagocytic assay, 1×10^5^ per well WT-BMDM or KO-BMDM were seeded into 12-well plates. After the BMDMs adhesion, Raji and K562 cells labeled using carboxyfluorescein diacetate succinimidyl ester (CFSE) were added and incubated with BMDMs for 1 hour at 37°C. Then, all cells were collected and stained with APC-CD11b antibody for 20 minutes at 4°C in the dark and analyzed using Flow Cytometry. Additionally, when BMDMs are infected with recombinant adeno-associated virus (rAAV), the Raji or K562 cells are then labeled with CellTrace™ Far Red (APC channel, Thermo Fisher Scientific, #C34572, USA), and all cells were collected and stained with PE-CD11b.

### Recombination Adeno-Associated Virus (rAAV)

The triple plasmid system (the vector pscAAV-CAG-GFP (Addgene, #83279) or pscAAV-CAG-CYPJ or pscAAV-CAG-mutCYPJ, the serotype AAV1 Rep/Cap (Addgene, #112862) and helper plasmid (Addgene, #112867) were used for rAAV production. The specific packaging method is as described previously 25. Briefly, 293T cells were transfected with the triple plasmid system at a molar ratio of 1:1:1 using PEI (Yeasen, #40816ES02). After 72 h or 96 h, the supernatant was used to precipitate the virus with 40% PEG 8000 (Beyotime, #ST483), and the cells were subjected to three cycles of freeze-thawing to release the virus. Ultimately, all the viruses were pooled, and after undergoing iodixanol gradient density centrifugation and ultrafiltration concentration, a high-titer purified rAAV virus was obtained for infection of BMDMs.

### Western Blotting (WB) and Co-Immunoprecipitation (Co-IP)

For the western blotting, cells were lysed using a lysis buffer (50 mM Tris-HCl, pH 7.4, 150 mM NaCl and 0.1% Triton X-100) with protease inhibitor cocktail (TargetMol, #C0001, USA) for 30 minutes. Then, supernatants were collected after centrifugation at 12,000 rpm at 4 °C for 10 minutes. Next, add the protein loading buffer and boil for 5 minutes for further SDS-PAGE analysis.

For the co-immunoprecipitation assay, the expression plasmids for CYPJ and AKT1 were obtained from the laboratory collection. The transfected cells were washed with PBS three times and then lysed with the IP lysis buffer (Cell Signaling Technology, #9803S, USA) for 30 minutes. At the same time, the Protein A/G Immunomagnetic Beads (TargetMol, #C0104B, USA) were incubated with antibodies for 15 minutes at room temperature. Then, a cellular supernatant was added. After incubation overnight at 4 °C, the beads were washed three times, and the following steps were the same as WB. All antibody information is recorded in **Supplementary Table 2**.

### Human Samples and Multiplex Immunohistochemistry (mIHC)

Sixty-one pairs of LIHC and adjacent normal paraffin-embedded tissue chips were collected from Sun Yat-Sen University Cancer Center (SYSUCC). All tissue samples were approved by the Medical Ethical Committee of the SYSUCC (Approval number: G2023-019-01).

The tissue chips were stained by a Panovue 6-color multiplex immunohistochemical staining kit (Panovue, #10234100020), and the specific steps were carried out according to the actual instructions. In brief, the target genes were labeled with a primary antibody (CD86, CD163, CD8, CYPJ, and EpCAM), followed by a reaction with the secondary antibody in the kit. Then, the fluorescence was marked using tyramide signal amplification (TSA) technology. After the staining was completed, the slides were scanned using the Vectra Polaris™ Automated Quantitative Pathology Imaging System (Akoya Biosciences, USA), and the data was analyzed by the pathology analysis software HALO (v2.0, Indica Labs, USA).

In fluorescence-labeled cells, DAPI staining-positive areas represent the total number of cells in each sample, and then other markers were defined as positive cells according to the fluorescence intensity. HALO software automatically calculated the number of labeled positive cells and divided this number by the total number of cells represented by DAPI-staining areas. Then the proportion of positive cells corresponding to fluorescent labeling in each sample was finally calculated. Immunohistochemistry (IHC) steps are similar to mIHC. All the relevant antibody information is recorded in **Supplementary Table 2**.

### Statistical Analysis

GraphPad Prism 8.0 was used for data analyses and visualization. The analysis between tumors and the adjacent tissues was performed using the paired t-test. The differences between the two groups were determined with the student's t-test. The analysis of the three groups and above used one-way ANOVA. The data of pathological grade of patients were analyzed by Yates'continuity corrected chi-square test. For all analyses, a p-value < 0.05 was considered statistically significant (*p < 0.05, **p < 0.01, ***p < 0.001, ****p < 0.0001, and ns = non-significant). All data represent the mean ± SD.

## Results

### CYPJ Inhibits the Progression of LIHC *in vivo*

**Figure 1:**
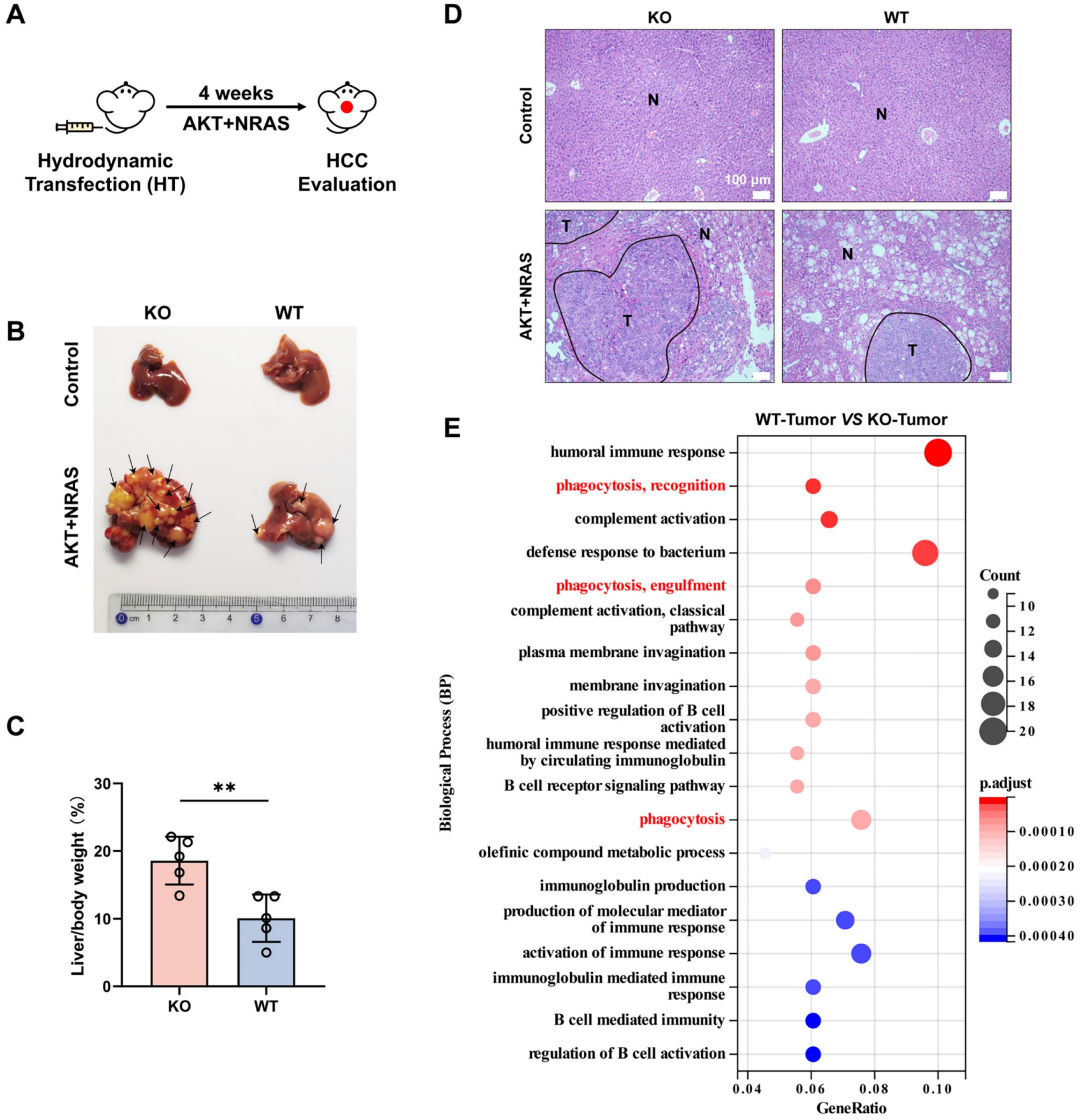
** CYPJ Inhibits the Progression of LIHC *in vivo*.** (**A**) Strategy for hydrodynamic transfection-induced liver cancer model. Male mice received a rapid tail vein injection of 20 μg AKT1, 20 μg NRAS and 1.6 μg SB transposon within 7 seconds. (**B**) Representative images of tumor generation in the liver of wild-type mice (WT group) and *Cypj*-deficient mice (KO group). (**C**) Percentage of liver weight ratio (liver/body weight) in WT and KO mice, respectively. (**D**) Representative H&E staining of liver tissues harvested from both WT and KO groups. Tumor (T) and Normal (N) tissue are outlined, respectively. Scale bars, 100 μm. (**E**) Biological process enrichment pathway analyses of liver tumors between wild-type mice (WT-Tumor) and *Cypj*-deficient mice (KO-Tumor). (*p <0.05, ** p <0.01, *** p <0.001, **** p <0.0001, ns=non-significant).

Our previous study found that CYPJ is a suppressor of colorectal cancer (CRC). To explore whether CYPJ also plays a tumor suppressor role in other tumors, we conducted a mouse primary liver cancer model generated by hydrodynamic transfection technology. Co-expression with multiple potent oncogenes such as AKT and NRAS (G12V) could efficiently lead to the generation of liver cancer in male mice (**Figure 1A**). We carried out hydrodynamic transfection experiments on wild-type mice (WT group) and *Cypj*-deficient mice (KO group), and the results showed that the knockout of* Cypj* led to more severe liver cancer (**Figure 1B**). The liver weight ratio in the KO group was dramatically higher than that in the WT group, and the tumor number and tumor area in the liver of KO mice were much greater than those in the WT counterparts (**Figure 1C, D**).

Next, the survival analysis of LIHC on the Kaplan-Meier Plotter website revealed that the higher expression of CYPJ correlated with a better prognosis (**Supplementary Figure 1A**). These results indicated that CYPJ was an inhibitor in the progression of liver cancer. To further investigate the mechanism underlying CYPJ's inhibitory effect on LIHC, we sent the liver tumor tissues of the KO group and WT group for RNA-Seq, and the biological process analysis suggested that the immune-related pathways were significantly activated in the WT group, especially the phagocytosis related to macrophages (**Figure 1E**). Furthermore, we analyzed the distribution of CYPJ in immune cells of several cancers that CYPJ was highly expressed in tumors on the TISCH1 website, and the single-cell data showed that the expression of CYPJ was enriched in TAMs and T cells (**Supplementary Figure 1B**). Then, the analysis of immune cell infiltration in LIHC showed that the expression of CYPJ was positively correlated with M1 TAMs infiltration using the CIBERSORT-ABS algorithm (**Supplementary Figure 1C**). Also, the survival analysis based on the TIMER2.0 website showed that patients with higher levels of CYPJ and higher proportions of infiltrating macrophages had better survival in LIHC (**Supplementary Figure 1D**). Overall, these findings suggested that the tumor inhibition role of CYPJ in LIHC is associated with the function of TAMs.

### CYPJ Promotes the Infiltration of M1 TAMs and CD8^+^ T Cells toward Anti-tumor Effects

**Figure 2:**
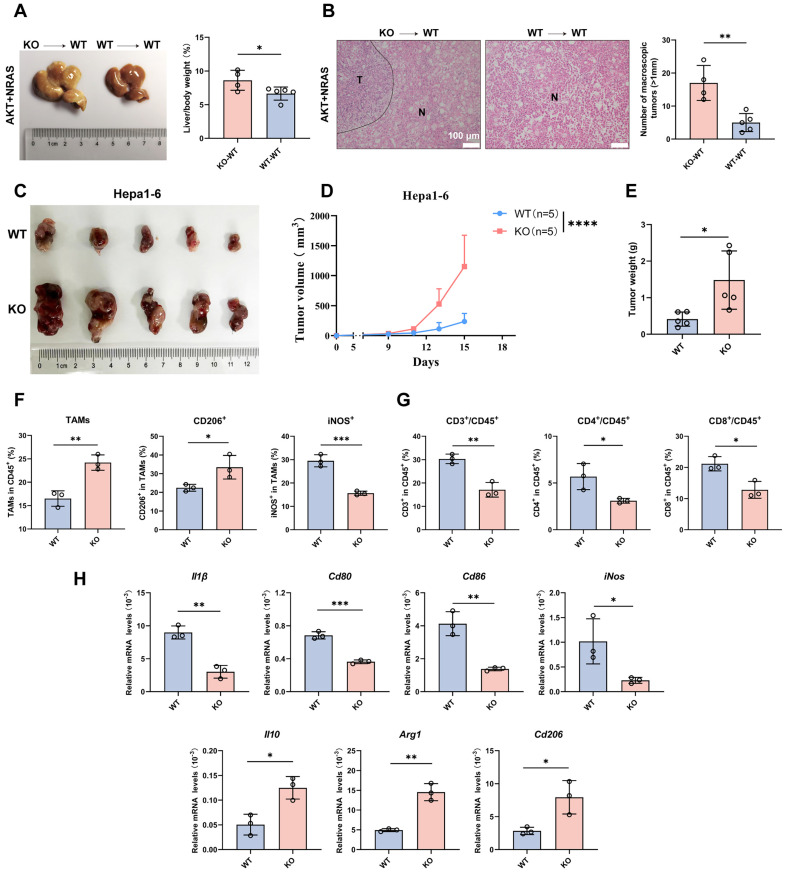
** CYPJ Inhibits the Development of LIHC by Promoting the Infiltration of M1 TAMs and CD8^+^T cells.** (**A**) Liver tumor and liver weight ratio in bone marrow chimera: KO→WT chimera (bone marrow-derived from KO mice injected into WT recipients), and WT→WT chimera (bone marrow-derived from WT mice injected into WT recipients). (**B**) Representative H&E staining of liver tissues harvested from bone marrow chimera includes KO→WT chimera and WT→WT chimera. Tumor (T) and Normal (N) tissue are outlined, respectively. Scale bars, 100 μm. (**C**) Subcutaneous tumor formation of Hepa1-6 cells in WT and KO mice (1×10^6^ Hepa1-6 cells per mouse). (**D, E**) Tumor growth curve (**D**) and final tumor weight (**E**) of Hepa1-6 tumors in WT and KO mice. (**F**) Flow cytometry analysis of TAMs. The TAMs are classified by CD45^+^/CD11b^+^/F4/80^+^ cells and normalized in CD45^+^ cells. The anti-tumor M1 TAMs were detected as CD45^+^/CD11b^+^/F4/80^+^/iNOS^+^ cells, whereas tumor-promoting M2 TAMs were identified as CD45^+^/CD11b^+^/F4/80^+^/CD206^+^ cells. (**G**) Flow cytometry analysis of T cells defined as CD45^+^/CD3^+^/CD4^+^ T cells and CD45^+^/CD3^+^/CD8^+^ T cells. The proportion of CD4^+^ or CD8^+^ T cells was normalized in CD45^+^ cells. (**H**) qRT-PCR detect the expression of M1 markers (*Il1β*, *Cd80*, *Cd86* and *iNos*) and M2 markers (*Il10*, *Arg1* and *Cd206*). (*p <0.05, ** p <0.01, *** p <0.001, **** p <0.0001, ns=non-significant).

As CYPJ inhibits the development of LIHC, we further explored whether the function of CYPJ is related to the immune system, particularly in terms of TAMs. Firstly, we conducted hydrodynamic transfection experiments after establishing the bone marrow chimera (**Supplementary Figure 2A**). Then, significant differences were observed between the KO→WT chimera (bone marrow-derived from KO mice injected into WT recipients) and WT→WT chimera (bone marrow-derived from WT mice injected into WT recipients), with the development of tumors being more severe in the KO→WT chimera (**Figure 2A, B**). Meanwhile, subcutaneous tumor formation experiments in WT and KO mice were performed to explore the relationship between CYPJ and immune cells by injecting the murine Hepa1-6 cell line. We observed that the tumor size (**Figure 2C**), tumor volume (**Figure 2D**) and tumor weight (**Figure 2E**) of WT mice were much smaller than KO counterparts. Additionally, by flow cytometry analysis of TAMs and T cells in the subcutaneous tumor, we found that the number of TAMs was less in the WT group compared to the KO group (**Figure 2F**). However, the WT group had a higher proportion of anti-tumor M1 TAMs, while the KO group had a higher proportion of pro-tumor M2 TAMs (**Figure 2F, Supplementary Figure 2B**). In the analysis of T cells, the percentage of both CD4^+^ and CD8^+^ T cells was lower in the KO group than in the WT group (**Figure 2G, Supplementary Figure 2C**). Moreover, we extracted RNA from subcutaneous tumor tissues and detected markers of TAMs through the qRT-PCR. The results showed that markers of M1 TAMs in the WT group, such as* Il1β*, *Cd80*, *Cd86* and *iNos*, were significantly elevated (**Figure 2H**). In contrast, markers associated with M2 TAMs in the KO group, such as *Il10*, *Arg1* and *Cd206*, were increased (**Figure 2H**). These results demonstrated that high expression of CYPJ promoted the infiltration of M1 macrophages and CD8^+^ T cells in LIHC, thereby exerting an anti-tumor effect.

### CYPJ Promotes the Polarization of Macrophages towards an M1 Phenotype *in vitro*

**Figure 3:**
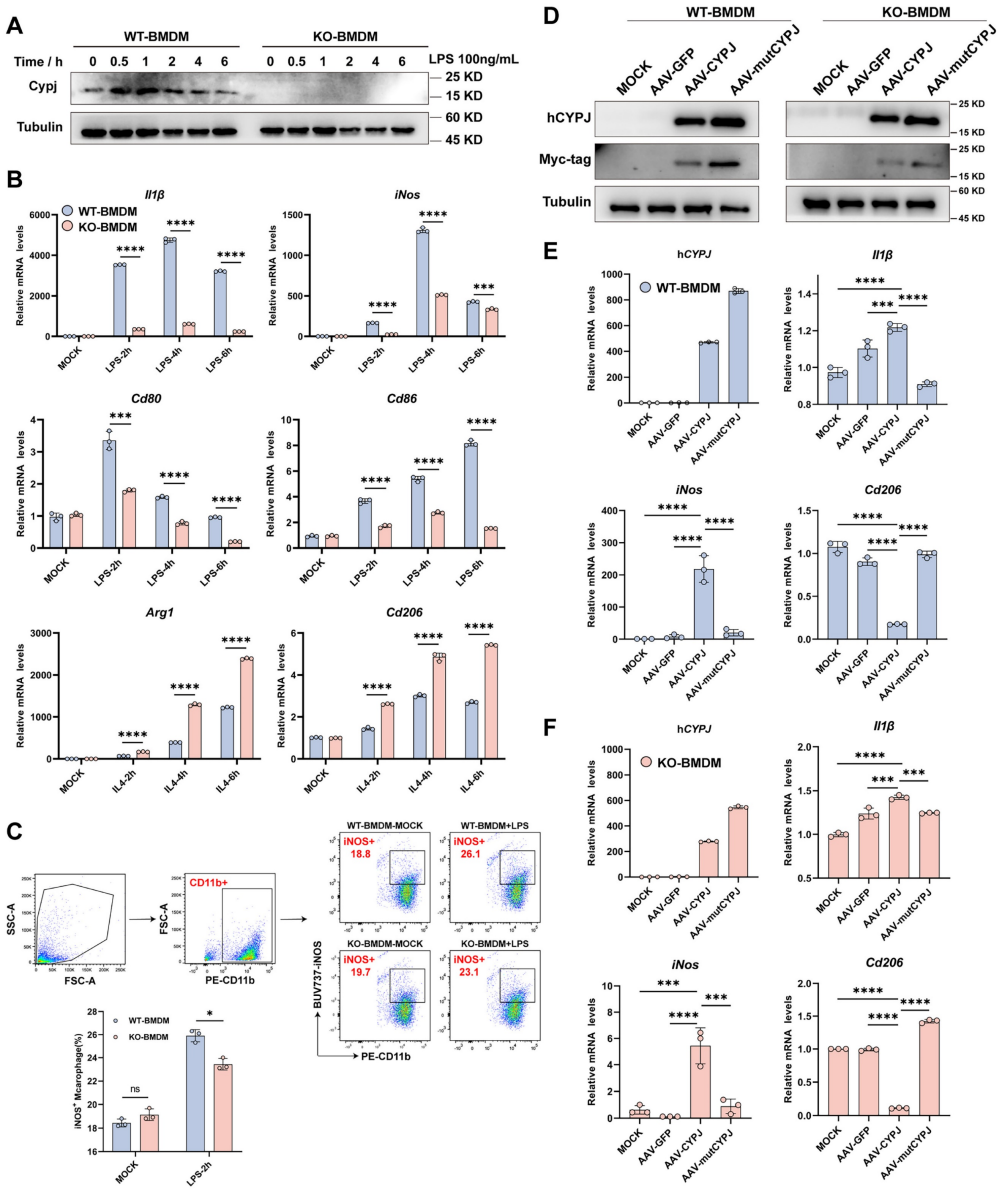
** CYPJ Drives Macrophage Polarization to the M1 Phenotype *in vitro*.** (**A**) Expression of CYPJ in WT and KO-BMDM under the stimulation of LPS (the classic M1 macrophage inducer). (**B**) qRT-PCR analysis to detect M1 markers (*Il1β*, *Cd80*, *Cd86* and *iNos*) and M2 markers (*Arg1* and *Cd206*) in WT and KO BMDM stimulated with LPS (100 ng/mL) or IL4 (20 ng/mL) in different time points. (**C**) Flow cytometry analysis of the proportion of M1 macrophages under the stimulation of LPS. Using the CD11b labels BMDMs, iNOS^+^ cells were identified as M1 macrophages. (**D**) WB analysis to assess the expression of Myc-tagged CYPJ/mutCYPJ (conserved sequence of CYPJ in humans) in WT/KO-BMDM (Tubulin as an internal reference). (**E, F**) qRT-PCR analysis to measure the expression of h*CYPJ* (conserved sequence of CYPJ in humans), M1 (*Il1β* and* iNos*) or M2 (*Cd206*) markers in WT-BMDM (**E**) or KO-BMDM (**F**) after AAV infection. MOCK cells served as the negative control, which remained uninfected, while AAV-GFP/CYPJ/mutCYPJ groups were infected with the recombinant adeno-associated virus. (*p <0.05, ** p <0.01, *** p <0.001, **** p <0.0001, ns=non-significant).

Since we have demonstrated that *Cypj*-KO in immune cells attenuates the infiltration of M1 TAMs in tumors, we further hypothesized whether CYPJ can directly regulate macrophages to function as a tumor suppressor. Subsequently, we conducted a series of experiments *in vitro*. Our results indicated that the expression of CYPJ in BMDMs from wild-type mice can be upregulated to increase under the stimulation of LPS (the classic M1 macrophage inducer) (**Figure 3A**). Additionally, the upregulation of M1 markers (*Il1β*, *iNos, Cd80* and *Cd86*) was more pronounced in WT-BMDM compared to KO-BMDM upon LPS stimulation (**Figure 3B**), and the response of KO-BMDM to M2 markers (*Arg1* and *Cd206*) was greater than that of WT-BMDM under stimulation by IL4 (the classic M2 macrophage inducer, **Figure 3B**). Besides, we confirmed that stimulation with LPS promoted a greater polarization of WT-BMDM towards the M1 phenotype compared with KO-BMDM by using flow cytometry detection (**Figure 3C**). In addition, we also stimulated WT-BMDM or KO-BMDM with the conditioned medium derived from Hepa1-6 cells (Hepa1-6 CM) and similar results could also be observed. The results showed that the stimulation with Hepa1-6 CM increased the levels of CYPJ and promoted WT-BMDM towards the M1 phenotype compared with KO-BMDM (**Supplementary Figure 3A, B, C**).

To further clarify the regulation of macrophages by CYPJ, we constructed CYPJ and its enzyme inactive mutant (mutCYPJ, with mutations at R44A and F49A). Due to the highly conserved amino acid sequence of CYPJ in humans and mice (**Supplementary Figure 3D**), we directly over-expressed human CYPJ (hCYPJ) and mutCYPJ in WT-BMDM or KO-BMDM using recombinant adeno-associated virus (rAAV) (**Figure 3D**). Subsequently, we found that over-expression of CYPJ promoted the upregulation of M1 markers (*Il1β* and *iNos*) and inhibited the levels of M2 markers (*Cd206*) in both WT-BMDM and KO-BMDM (**Figure 3E, F, Supplementary Figure 3E**). And the phenomenon was dependent on the peptidyl-prolyl cis-trans isomerase (PPIase) activity of CYPJ (**Figure 3E, F, Supplementary Figure 3E**). Besides, as cyclophilins were a group of proteins with PPIase activity, we also generated rAAV viruses infected WT-BMDM with another member of the cyclophilins, CYPA and its enzyme inactive mutant (mutCYPA at R55A and F60A). Similar to the findings of CYPJ, our data indicated that CYPA also up-regulated M1 markers (*Il1β* and *iNos*) and the function was also dependent on the PPIase activity of CYPA (**Supplementary Figure 3F**). The results suggested that the cyclophilins have similar functions in the regulation of macrophage polarization. Taken together, these data supported the hypothesis that CYPJ can directly regulate the polarization of macrophages towards an M1 phenotype which plays an anti-tumor role.

### CYPJ Promotes the Phagocytosis of Macrophages for Inhibiting Tumor Progression

**Figure 4:**
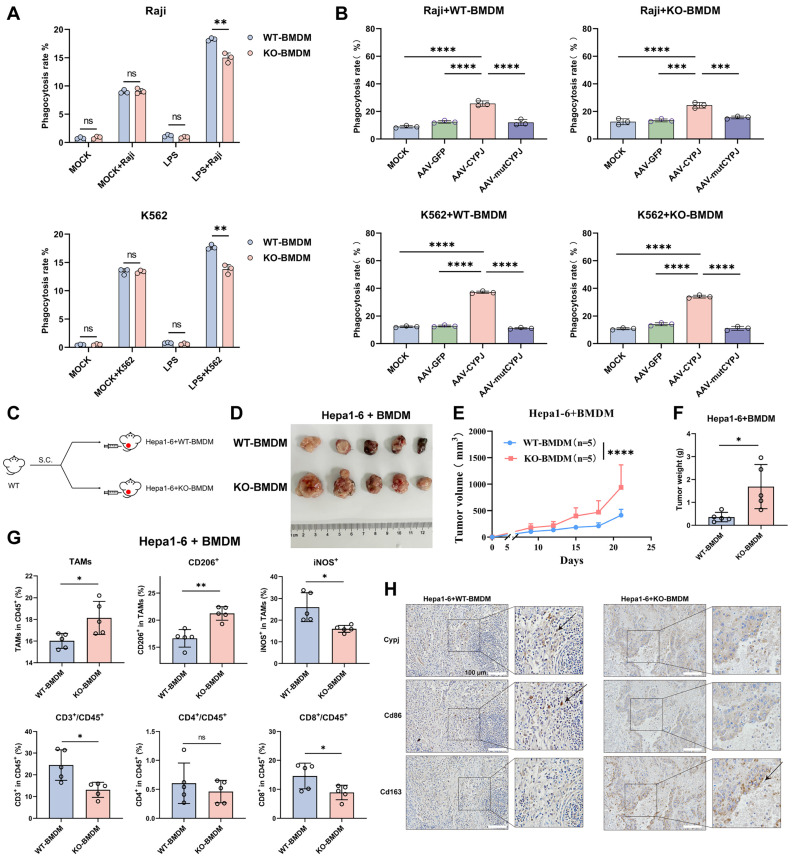
** CYPJ Promotes the Phagocytosis of Macrophages *in vitro* and exerts an Anti-Tumor Effect *in vivo*.** (**A**) Macrophage phagocytosis to Raji and K562 (labeled with CFSE) in WT/KO-BMDM under LPS stimulation. BMDMs and Raji cells were incubated at a 1:2 ratio for 1 hour at 37 ℃, while K562 was incubated at a 1:3 ratio for 1 hour at 37 ℃. (**B**) Phagocytosis of WT/KO-BMDM to Raji (at a 1:3 ratio) or K562 (at a 1:4 ratio) cell for 1 hour at 37 ℃ after AAV infection. (**C, D**) Strategy and tumor formation for subcutaneous tumors of Hepa1-6 cells mixed with WT-BMDM or KO-BMDM in percentage 1:1, a total of 1 × 10^6^ cells. (**E, F**) Tumor growth curve (**E**) and final tumor weight (**F**) of Hepa1-6 tumors mixed with WT-BMDM or KO-BMDM. (**G**) Flow cytometry analysis of TAMs and T cells within tumors. Gating strategies were consistent with those shown in Supplementary Figure 2B and 2C. (**H**) Representative IHC staining for murine Cypj, Cd86 (M1 marker), and Cd163 (M2 marker) in consecutive sections. Scale bar, 100 μm. (*p <0.05, ** p <0.01, *** p <0.001, **** p <0.0001, ns=non-significant).

As the engulfment is an essential function of macrophages for tumor elimination, we performed phagocytosis assays and noticed that WT-BMDM was able to engulf more Raji and K562 tumor cells under LPS stimulation (**Figure 4A, Supplementary Figure 4A, B**). Additionally, both WT-BMDM and KO-BMDM showed a significantly enhanced ability to engulf tumor cells after overexpressed CYPJ in BMDMs, but the phenomenon was not observed in the mutCYPJ group (**Figure 4B, Supplementary Figure 4C, D**). Next, we conducted a subcutaneous tumor experiment mixing Hepa1-6 cells with WT-BMDM or KO-BMDM to explore how CYPJ in macrophages affects tumor progression (**Figure 4C**). The results revealed that the tumors of Hepa1-6 cells mixed with WT-BMDM were much smaller in volume and weight than those mixed with KO-BMDM (**Figure 4D, E, F**). Flow cytometry analysis of immune cell infiltration also suggested that the group mixed with WT-BMDM had more iNOS^+^ TAMs (M1 TAMs) and CD8^+^ T cells (**Figure 4G**). The qRT-PCR detection of macrophage-related markers indicated that the expression of M1-related markers, such as* Il1β*, *Cd80*, *Cd86* and *iNos*, was increased in the group mixed with WT-BMDM (**Supplementary Figure 4E**). Additionally, we examined the expression of murine Cypj, Cd86 (M1 TAMs) and Cd163 (M2 TAMs) in Hepa1-6 tumors by using immunohistochemical (IHC) reactions and multiplex immunohistochemistry (mIHC). The pictures showed that tumors mixed with WT-BMDM had higher expression of Cypj and Cd86 (**Figure 4H, Supplementary Figure 5**), while tumors mixed with KO-BMDM showed increased levels of Cd163 (**Figure 4H, Supplementary Figure 6**). Taken together, the phagocytosis assays and subcutaneous tumor experiment further confirmed that CYPJ in macrophages boosts phagocytosis, which is crucial for antitumor immunity.

### CYPJ Inhibits the AKT1 Signal Pathway to Promote M1 Phenotype of Macrophages

**Figure 5:**
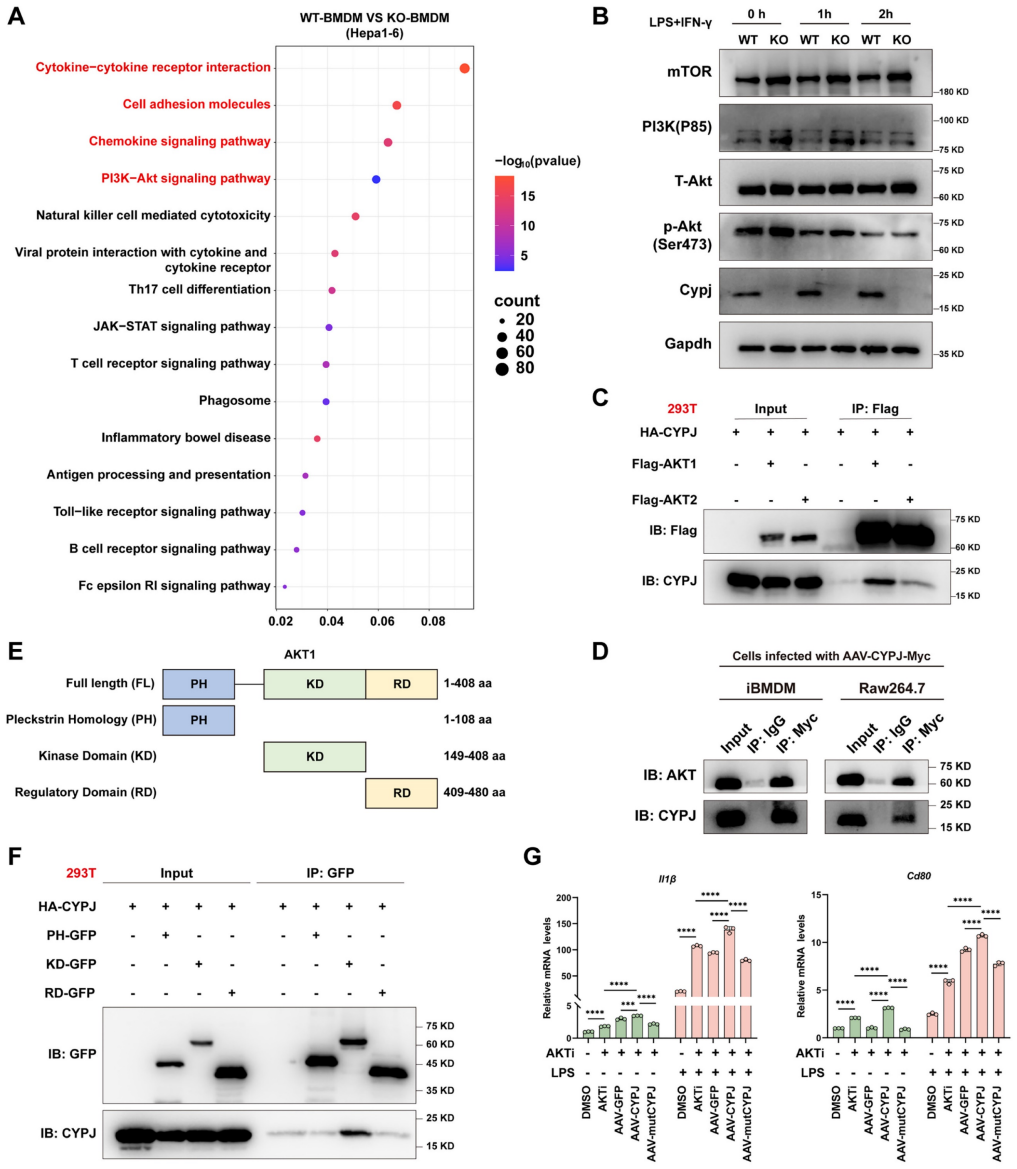
** CYPJ Interacts with AKT1 and promotes Macrophage Polarization to the M1 Phenotype.** (**A**) KEGG enrichment analysis of Hepa1-6 cells mixed with WT-BMDM or KO-BMDM. (**B**) WB analysis of PI3K-AKT signal pathway in WT-BMDM and KO-BMDM under stimulation of M1 macrophage inducer. (**C**) WB analysis of interactions between CYPJ and AKT1/2 determined by co-immunoprecipitation with anti-Flag in 293T cells. HA-CYPJ and Flag-AKT1/2 expression plasmids were co-transfected into 293T cells. (**D**) WB analysis of interactions between CYPJ and AKT determined by co-immunoprecipitation with anti-Myc in iBMDM and RAW264.7 cells. Whole-cell lysates of iBMDM and RAW264.7 infected with AAV-CYPJ-Myc were collected, with IgG as a control group. (**E**) A schematic diagram of the various domains in AKT1. FL, full length; PH, pleckstrin homology; KD, kinase domain; RD, regulatory domain. (**F**) Co-immunoprecipitation assay between CYPJ and different domains of AKT1. HA-CYPJ and PH/KD/RD-GFP expression plasmids were co-transfected into 293T cells, which were then subjected to IP with an anti-GFP antibody. (**G**) qRT-PCR analysis to measure the expression of M1 markers (*Il1β* and* Cd80*) in WT-BMDM or KO-BMDM after treatment of AKT inhibitor (AKTi, MK2206, 5 μM pre-treat for 24 h) and AAV-GFP/CYPJ/mutCYPJ. DMSO cells served as the negative control. (*p <0.05, ** p <0.01, *** p <0.001, **** p <0.0001, ns=non-significant).

To uncover the mechanism of CYPJ in regulating macrophage polarization, we subjected tumors mixed with Hepa1-6 cells and either WT-BMDM or KO-BMDM to RNA-seq. The results of KEGG pathway enrichment analysis showed that numerous signal pathways related to immune regulation could be enriched, such as cytokine-related signaling pathways (**Figure 5A**), notably a marked increase in markers associated with M1 polarization in WT-BMDM group (**Supplementary Figure 7A, Supplementary Table 3**), as well as anti-tumor chemokines such as Cxcl9 and Cxcl10 (**Supplementary Figure 7B, Supplementary Table 4**). Cxcl9 and Cxcl10 are known to recruit and activate CD8^+^ T cells, enabling their synergistic tumoricidal activity with M1 macrophages 26, 27. Consistent with this, we observed increased infiltration of CD8^+^ T cells in tumors, suggesting that CYPJ may promote cytokines secretion by macrophages, thereby recruiting CD8^+^ T cells to synergize with M1 TAMs to exert an anti-tumor effect.

Moreover, the RNA-Seq analysis also found significant enrichment of the PI3K-AKT signal pathway, which has been reported to be closely related to macrophage polarization (**Figure 5A**). Therefore, we speculated that CYPJ may regulate the macrophage polarization through the PI3K-AKT signal pathway. It is reported that the regulation of macrophage polarization by the PI3K-AKT pathway is dualistic 28. On the one hand, AKT can enhance the activation of mTORC1, thereby promoting the polarization of macrophages towards the M1 phenotype 29. On the other hand, the activation of PI3K-AKT predominantly promotes the polarization of macrophages towards the M2 phenotype through various mechanisms 30-32. In our study, the levels of mTOR, PI3K, and phosphorylated AKT (p-AKT) in WT-BMDMs were suppressed compared to KO-BMDM when induction of M1 polarization (**Figure 5B**). This was also confirmed by re-introducing CYPJ in KO-BMDM, where p-AKT was significantly suppressed (**Supplementary Figure 7C**). Subsequently, through co-immunoprecipitation experiments, we found that CYPJ had a strong interaction with AKT1 in 293T cells, iBMDM and RAW264.7 macrophages (**Figure 5C, D**). Further experiments confirmed that CYPJ interacted with the kinase domain of AKT1 (**Figure 5E, F**). Therefore, we assumed that CYPJ bound to the kinase domain of AKT1, disturbing its activation and thereby suppressing the macrophage polarization towards the M2 phenotype.

Furthermore, we treated BMDMs with an AKT inhibitor (MK2206) which resulted in the upregulation of M1 markers (*Il1β* and* Cd80*) and downregulation of M2 markers (*Arg1* and* Cd206*) following LPS or IL4 stimulation (**Supplementary Figure 7D**). Next, we pre-treated KO-BMDM with AKT inhibitor for 24 h before AAV-CYPJ supplementation (**Supplementary Figure 7E**). The results indicated that combined treatment with both AKT inhibitor and CYPJ yielded a greater increase in M1 markers (*Il1β and Cd80*) compared to individual treatments (**Figure 5G**). These findings further confirm that AKT inhibition promotes macrophage polarization toward the M1 phenotype and reveals a synergistic effect between AKT inhibition and CYPJ expression. Collectively, these results elucidate the molecular mechanism by which CYPJ regulates macrophage polarization.

### CYPJ is a Therapeutic Target for LIHC

**Figure 6:**
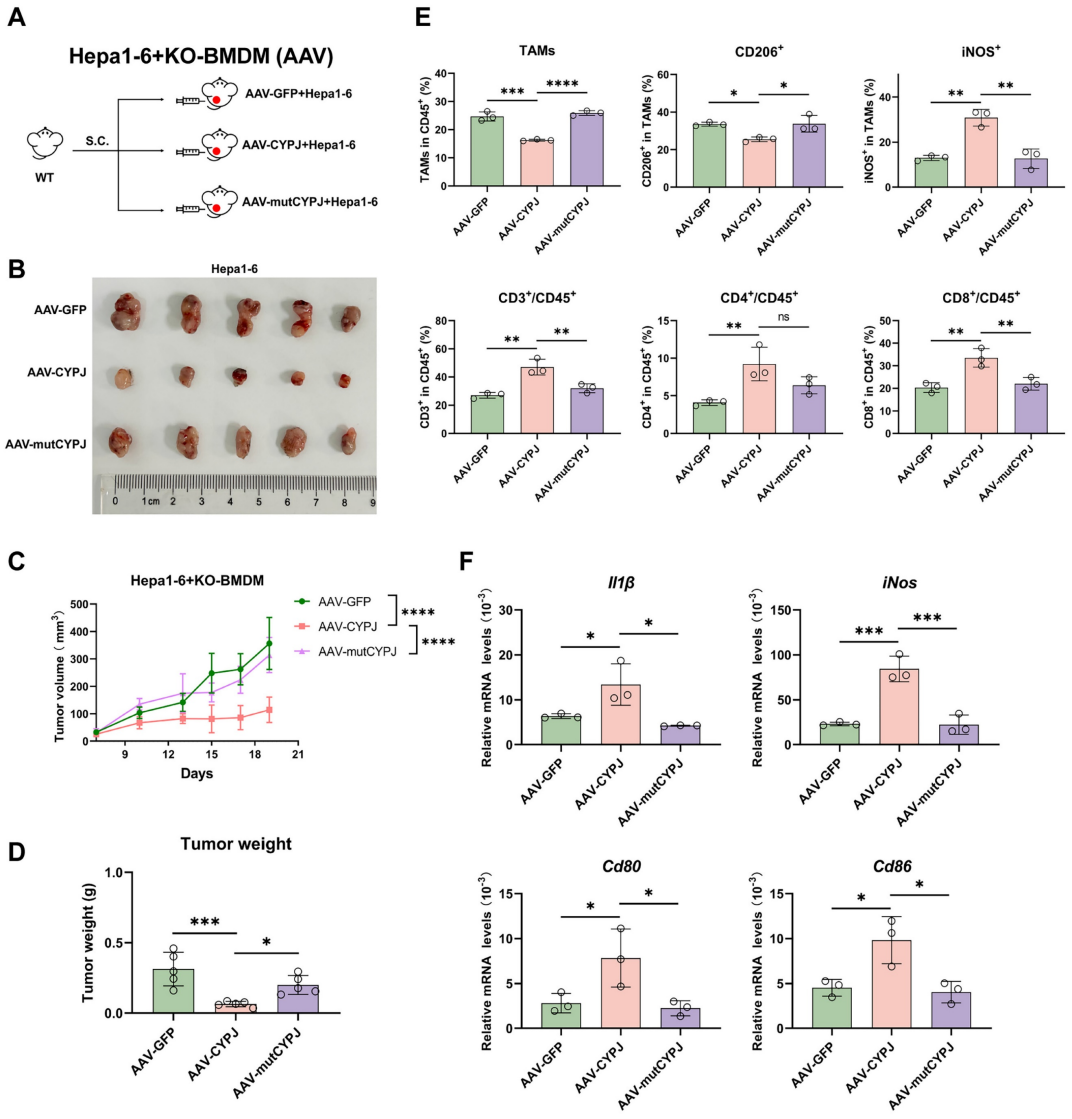
** CYPJ is a Therapeutic Target for LIHC.** (**A, B**) Strategy and tumor formation for subcutaneous tumors of Hepa1-6 cells mixed with KO-BMDM infected with different rAAV. The Hepa1-6 and KO-BMDM were mixed at a percentage 1:1 ratio, a total of 1 × 10^6^ cells. (**C, D**) The final tumor weight (**C**) and tumor growth curve (**D**) of Hepa1-6 cells mixed with KO-BMDM infected with AAV-GFP/CYPJ/mutCYPJ. (**E**) Flow cytometry analysis of TAMs and T cells in tumors. Gating strategies were the same as in Supplementary Figure 2B and 2C. (**F**) qRT-PCR detects M1 markers (*Il1β*, *Cd80*, *Cd86,* and *iNos*) in subcutaneous tumors of Hepa1-6 cells. (*p <0.05, ** p <0.01, *** p <0.001, **** p <0.0001, ns=non-significant).

To further investigate whether CYPJ can serve as a potential therapeutic target for macrophage-based cancer therapy, we replenished CYPJ in KO-BMDM using the rAAV method and then mixed them with Hepa1-6 cells for subcutaneous injection into wild-type mice (**Figure 6A**). The results indicated that replenishing CYPJ in KO-BMDM significantly inhibited tumor growth, with smaller tumor volumes and weights compared to the control group (**Figure 6B, C, D**). Furthermore, analysis of immune cell infiltration revealed that the proportion of anti-tumor M1 TAMs and CD8^+^ T cells was significantly increased in the CYPJ-overexpressed group (**Figure 6E**). Detection of M1-related markers in tissue RNA also showed a significant upregulation in the CYPJ-overexpressed group but not its inactive mutant (**Figure 6F**).

**Figure 7:**
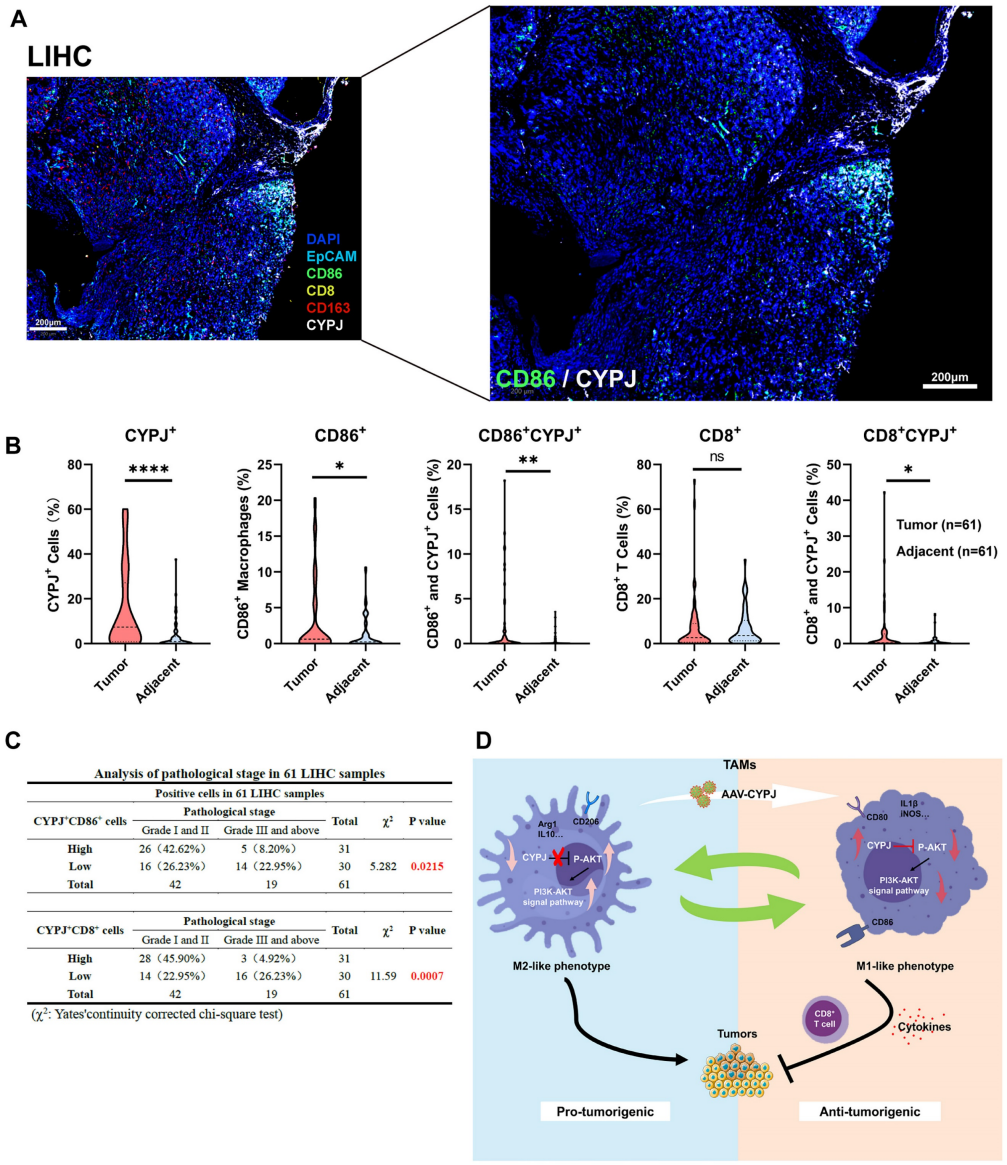
** Potential Influence of CYPJ in TAMs on Tumor Progression in LIHC Patients.** (**A**) mIHC of liver cancer tissue, labeled with DAPI (blue), CYPJ (white), CD86 (green), CD163 (red), CD8 (yellow), and EpCAM (cyan), then scanned using the Vectra Polaris Pathology Imaging System. Scale bar, 200 μm. (**B**) Quantification of positive cells for CYPJ, CD86, CD8, CD86^+^CYPJ^+^ and CD8^+^CYPJ^+^ in tumor tissues (n=61) and adjacent normal tissues (n=61) of the liver by mIHC. DAPI-stained areas indicate total cells. HALO software counted labeled positive cells, divided this by the DAPI-stained total cell count and calculated the proportion of positive cells. (**C**) Analysis of pathological stage in LIHC patients. The 61 tumor samples were divided into high and low groups according to the median of positive cells, then according to the pathological grade of the tumor, patients were divided into low grade (Grade I and II) and high grade (Grade III and above) groups, Chi-square test was used for analysis. (**D**) The proposed model for CYPJ reprograms macrophage toward an anti-tumorigenic M1 phenotype by inhibiting the AKT signal pathway. (*p <0.05, ** p <0.01, *** p <0.001, **** p <0.0001, ns=non-significant).

Ultimately, we checked if the expression of CYPJ in TAMs would affect the tumor progression in LIHC patients. We detected the expression of CYPJ, CD86 (a marker for M1 TAMs), CD163 (a marker for M2 TAMs), CD8 (a marker for CD8^+^ T cell), and EpCAM (a marker for tumor cells) in tissues from liver cancer patients using the mIHC method. The result indicated that CYPJ was co-expressed with CD86 in LIHC, and nearby CD8 expression was also elevated (**Figure 7A, Supplementary Figure 8**). Then, the analysis of CYPJ, CD86 and CD8 positive cells in 61 paired LIHC tumors and adjacent normal tissues showed that the positive cells of CYPJ and CD86 were significantly up-regulated in tumor tissue compared with adjacent normal tissues (**Figure 7B, Supplementary Figure 9**). Moreover, the percentage of CD86^+^CYPJ^+^ (M1 TAMs) macrophages and CD8^+^CYPJ^+^ T cells also increased in tumors (**Figure 7B**). Meanwhile, the 61 tumor samples were divided into high and low groups according to the median of positive cells. The results of the pathological stage analysis revealed that the patients with a higher proportion of CD86^+^CYPJ^+^ (M1 TAMs) macrophages or CD8^+^CYPJ^+^ T cells exhibited a lower pathological grade, indicating that CYPJ acted as a protective factor against liver cancer (**Figure 7C**). In summary, our findings demonstrated that CYPJ can reshape TAMs, promoting the polarization of TAMs towards an anti-tumor M1 phenotype and thereby exerting a tumor-killing effect in a PPIase activity-dependent manner. Therefore, CYPJ may serve as a therapeutic target for immunotherapy in liver cancer.

## Discussion

Macrophages play a complex role in the TME, and their role frequently hinges on their polarization state. In solid tumors, TAMs often exhibit an M2-like phenotype that promotes tumor development, whereas M1 TAMs inhibit tumor growth 33. Consequently, reprogramming macrophages towards the M1 type has emerged as a pivotal direction in macrophage-based anti-tumor therapy. Researchers have discovered that inhibiting YTHDF2 can reprogram TAMs to an anti-tumor phenotype and enhance their antigen cross-presentation ability to suppress tumor growth 34. Another study has proved that inhibiting APOC1 facilitates the transition of M2 to M1 macrophages via the ferroptosis pathway, enhancing the efficacy of anti-PD1 immunotherapy in liver cancer 35. In addition, the histone deacetylase inhibitor TMP195 has been shown to reprogram TAMs to an M1 phenotype, subsequently boosting their phagocytic activity in breast cancer 36. And in our study, we confirmed that CYPJ promotes the polarization of TAMs towards the M1 phenotype, enhances their phagocytic capacity, and activates CD8^+^ T cells to kill tumors. During this process, numerous cytokines are secreted to strengthen the communication between immune cells, and the entire TME is remodeled. These results indicate CYPJ is a new potential target for reprogramming TAMs.

Unlike direct TAMs depletion, TAM repolarization both decreases immunosuppressive TAMs and increases anti-tumor macrophages, more efficiently reversing the immunosuppressive TME. However, although promoting the transition from M2 to M1 holds great potential in cancer treatment, choosing the appropriate reprogramming method is important due to the limited understanding of polarization mechanisms *in vivo* and unpredictable immune responses within patients. Currently, the main reprogramming strategies encompass designing reprogrammed chimeric antigen receptor macrophages (CAR-M) or utilizing mRNA-carrying nanoparticle technology to reprogram macrophages. For example, Zhang and his colleagues introduced the intracellular TIR domain of TLR4 directly into the CAR to develop the second-generation CAR-M, which promotes an M1-like pro-inflammatory state, enhancing anti-tumor effects 37. Stephan et al. directly employed nanoparticles carrying mRNA of M1 polarization transcription factor, interferon regulatory factor 5, as well as its activating kinase IKKβ, to reprogram macrophages into a phenotype that induces anti-tumor immunity and promotes tumor regression 38. Kim and his team combined two technologies, directly injecting nanocomplexes composed of macrophage-targeting nanocarriers and CAR-interferon-γ-encoding plasmid DNA to induce CAR-M1 macrophages that are capable of CAR-mediated cancer phagocytosis 39. Unlike these studies, we selected AAV technology to reprogram macrophages directly in our research. AAV offers the advantages of high safety and low immunogenicity, and it can effectively transduce exogenous genes into macrophages 40, 41. In this study, we chose AAV serotype 1 to package CYPJ and directly infected BMDMs *in vitro*. Surprisingly, we successfully observed that AAV-CYPJ could promote the polarization of BMDMs towards the M1 phenotype and enhance their phagocytic ability. Furthermore, *in vivo* experiments demonstrated that macrophages edited by AAV-CYPJ could significantly inhibit tumor growth.

Although not very common, tumor suppressor genes can also be upregulated in tumors. For example, researchers have found that CXCL11 is highly expressed in colon cancer, which is associated with prolonged survival. Furthermore, the higher expression of the CXCL11, accompanied by a greater infiltration of anti-tumor immune cells such as CD8^+^ T cells, suggests that CXCL11 upregulation in tumors may stimulate immune cells to combat the tumor 42. In the present study, we also discovered that CYPJ is highly expressed in LIHC, and the high level of CYPJ is associated with an increase in M1 TAMs and CD8^+^ T cells, suggesting that the upregulation of CYPJ is a protective factor that limits tumor progression.

In conclusion, our study demonstrates that CYPJ acts as an inhibitor of tumor progression, particularly in LIHC. CYPJ promotes TAMs repolarization towards the M1 phenotype by inhibiting the activity of AKT1 and enhancing the innate and adaptive immune responses (**Figure 7D**), thereby facilitating the killing of tumor cells, which sheds light on the potential development of macrophage therapies for LIHC.

## Supplementary Material

Supplementary figures and tables.

## Abbreviations

TAMs: tumor-associated macrophages
TME: tumor microenvironment
LIHC: liver hepatocellular carcinoma
CYPJ: cyclophilin J
IFN-γ: interferon-γ
TNF: tumor necrosis factor
LPS: lipopolysaccharide
TGF: transforming growth factor
IL4: interleukin 4
M-CSF: macrophage colony-stimulating factor
iNOS: inducible nitric oxide synthase
PPIL3: peptidylprolyl isomerase like 3
CRC: colorectal cancer
SPF: specific pathogen-free
BMDMs: bone marrow-derived macrophages
CFSE: carboxyfluorescein diacetate succinimidyl ester
