# Supplementary material for: Cyclophilin J Reprograms Tumor-associated Macrophages to Exert an Anti-tumor Effect in Liver Cancer
Source: Int J Biol Sci. 2025 May 31;21(8):3776–90. doi: 10.7150/ijbs.113197 (PMC12160919; doi:10.7150/ijbs.113197)
Supplement: Supplementary file 1 — Supplementary figures and tables. [file ijbsv21p3776s1.pdf]

Supplementary Figures

Supplementary Figure 1

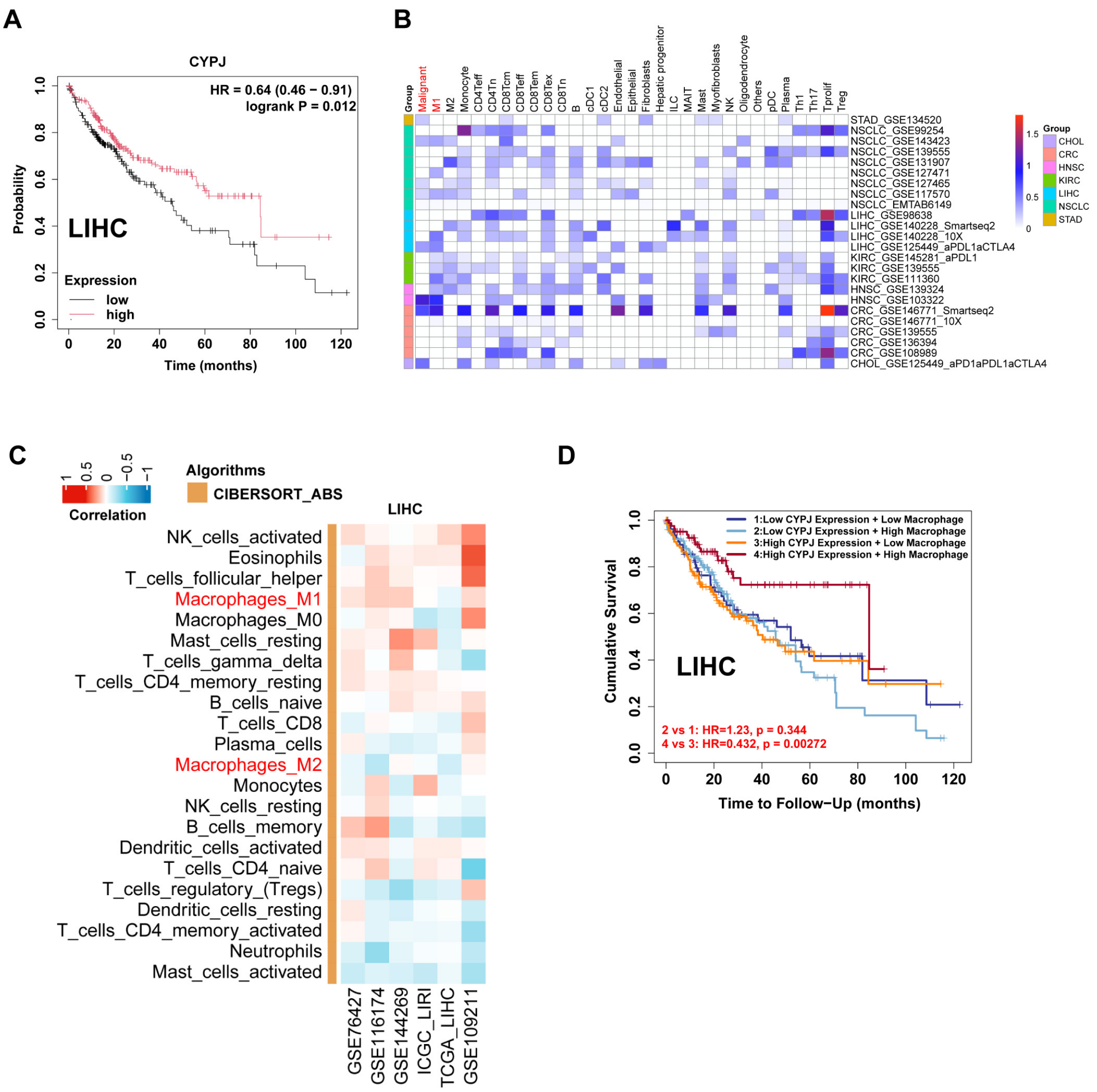

Supplementary Figure 1. CYPJ is Associated with M1 TAMs in LIHC.

(**A**) The survival analysis of CYPJ in LIHC was done on the Kaplan-Meier Plotter website. (**B**) Heatmap of CYPJ expression in the single cell expression of tumors with high CYPJ expression using the TISCH1 website. (**C**) Analysis of the relationship between immune cell infiltration and CYPJ using the CIBERSORT-ABS algorithm in the BEST website. (**D**) The survival analysis of CYPJ and macrophage in LIHC was done on the TIMER2.0 website.

Supplementary Figure 2

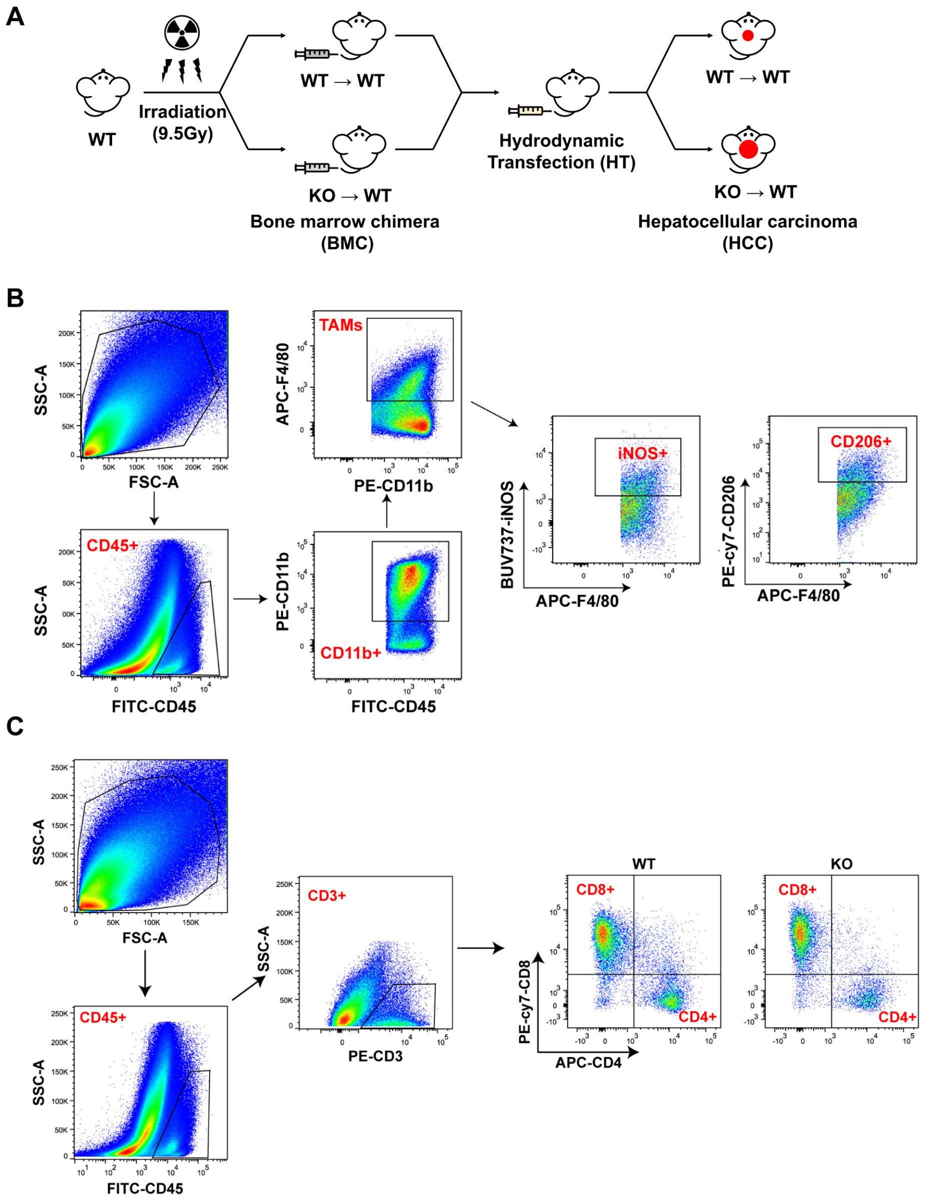

Supplementary Figure 2. The Strategy of Bone Marrow Chimera and Flow Cytometry Analysis of Immune Cell Infiltration in Tumors.

(**A**) Schematic diagram of bone marrow chimerism experiment. After feeding the recipient male mice with neomycin water, the recipient mice were lethally irradiated with 9.5 Gy. Bone marrow cells (5×10^6) harvested from the donor male mice were injected intravenously into irradiated recipients. (**B**) Gating strategy for TAMs in

tumors. Tumors were directly ground into single cells and resuspended in 3% BSA for staining. Immune cells were labeled with CD45 in tumors, and TAMs were defined by CD11b and F4/80. Then, $iNOS^+$ were identified as M1 TAMs, while $CD206^+$ were identified as M2 TAMs. (**C**) Gating strategy for T cells in tumors. Single cells are resuspended in 3% BSA, CD45 labels are used for immune cells, and CD3 labels total T cells. Further, T cells were defined as $CD4^+$ T cells and $CD8^+$ T cells.

Supplementary Figure 3

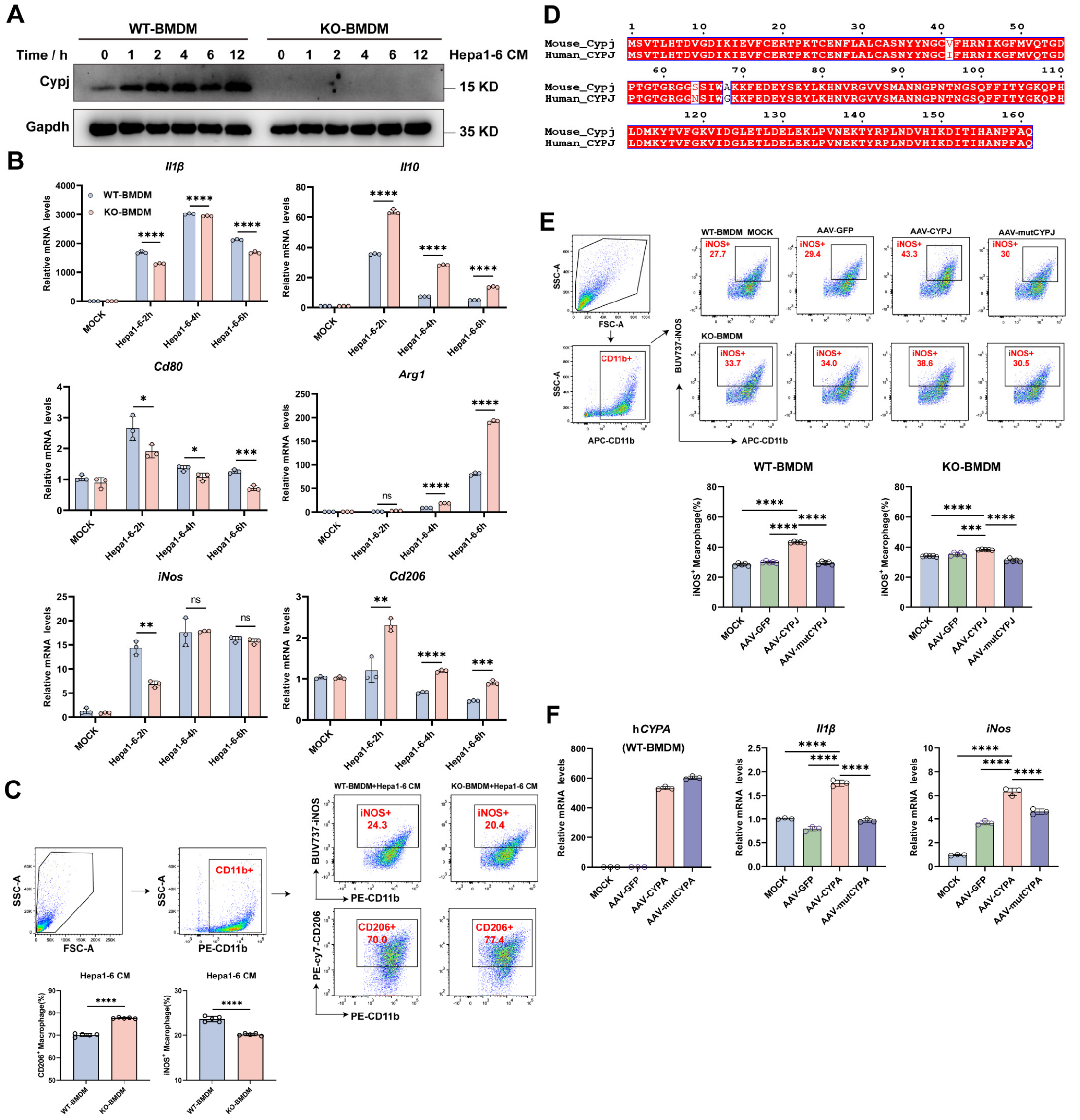

Supplementary Figure 3. CYPJ Promotes Macrophage Polarization to M1 Phenotype.

(**A**) Expression of CYPJ in WT-BMDM and KO-BMDM under the stimulation of tumor cell conditioned-medium (Hepa1-6 CM). (**B**) qRT-PCR detects M1(*Il1β, Cd80* and *iNos*) or M2 (*Il10, Arg1 and Cd206)* markers under Hepa1-6 CM stimulation. (**C**) Flow cytometry analysis of the proportion of M1 (iNOS^{+}) and M2 (CD206^{+}) macrophages with the stimulation of Hepa1-6 CM. (**D**) Comparative conservation analysis of the

amino acid sequence of CYPJ between humans and mice. (**E**) Gating strategy of macrophage polarization after AAV infection. The CD11b indicates BMDMs, and then the $iNOS^+$ cells were deemed to be M1 macrophages. (**F**) qRT-PCR analysis to measure the expression of hCYPA and M1 markers (*Il1β* and *iNos*) in WT-BMDM after AAV infection. MOCK cells served as the negative control. (*p <0.05, ** p <0.01, *** p <0.001, **** p <0.0001, ns=non-significant).

Supplementary Figure 4

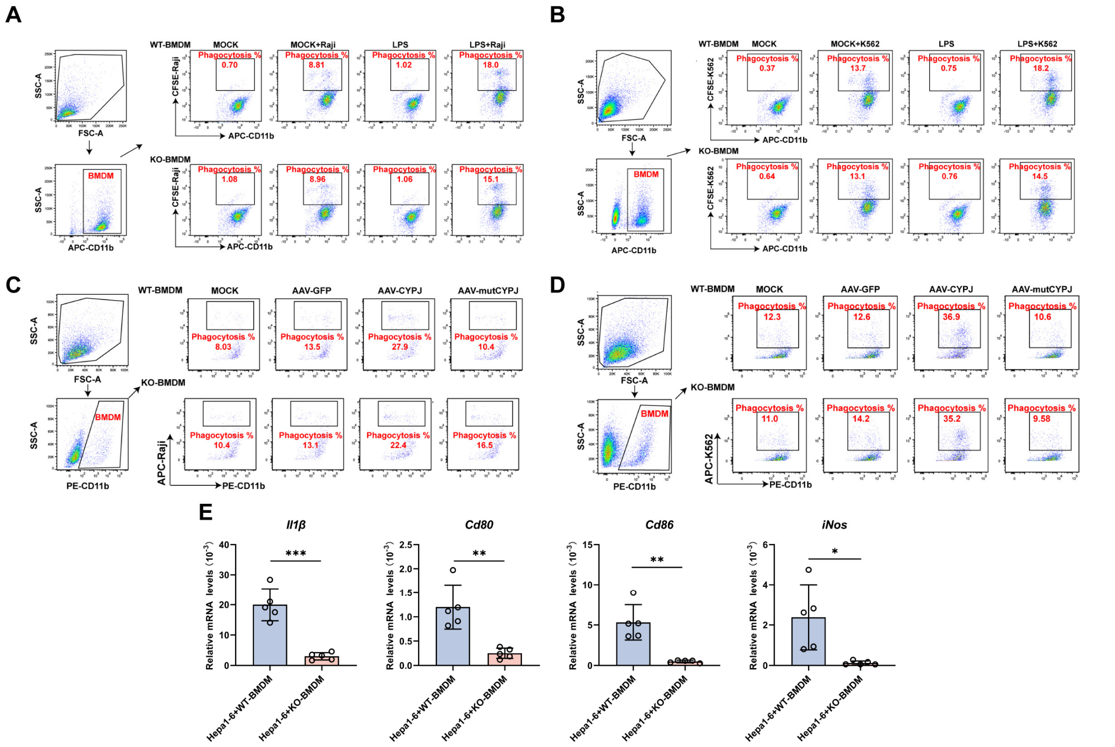

Supplementary Figure 4. Gating Strategy of Phagocytosis Experiments.

(**A, B**) Gating strategy of macrophage phagocytosis to Raji (**A**) and K562 (**B**) in WT/KO-BMDM under LPS stimulation. Raji and K562 are labeled with CFSE, and BMDMs are labeled with APC-CD11b. (**C, D**) Gating strategy of macrophages phagocytosis to Raji (**C**) and K562 (**D**) cells after AAV infection. Raji and K562 are labeled with CellTrace™ Far Red (APC channel), and BMDMs are labeled with PE-CD11b. (**E**) qRT-PCR detects M1 markers (*Il1β, Cd80, Cd86,* and *iNos*) in tumors of Hepa1-6 mixed with WT-BMDM or KO-BMDM. (*p <0.05, ** p <0.01, *** p <0.001, **** p <0.0001, ns=non-significant).

Supplementary Figure 5

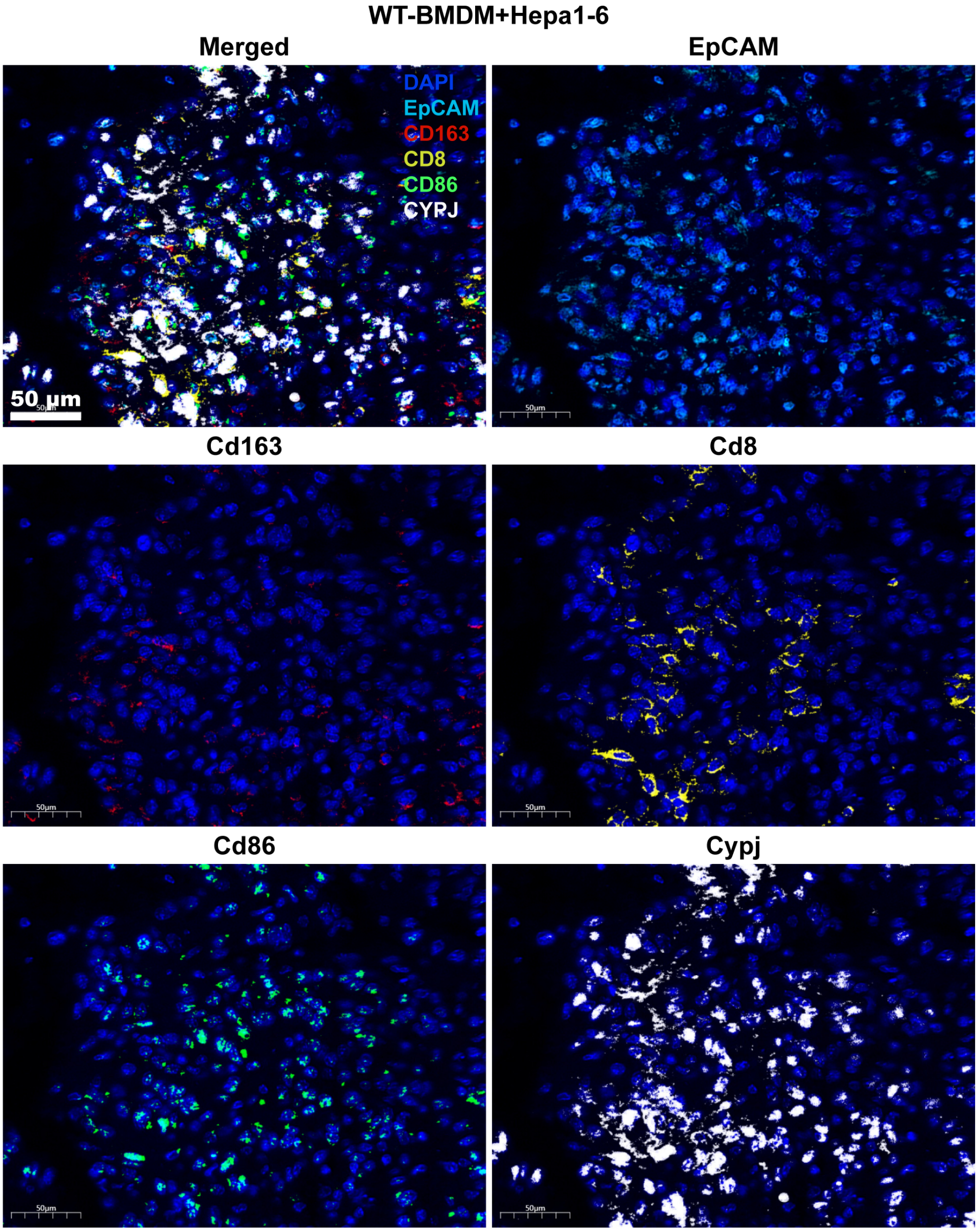

Supplementary Figure 5. Analysis of mIHC in Hepa1-6 Tumors Mixed with WT-BMDM.

mIHC of Hepa1-6 tumors mixed with WT-BMDM labeled with DAPI (blue), CYPJ (white), CD86 (green), CD163 (red), CD8 (yellow), and EpCAM (cyan). Scale bar, 50 µm.

Supplementary Figure 6

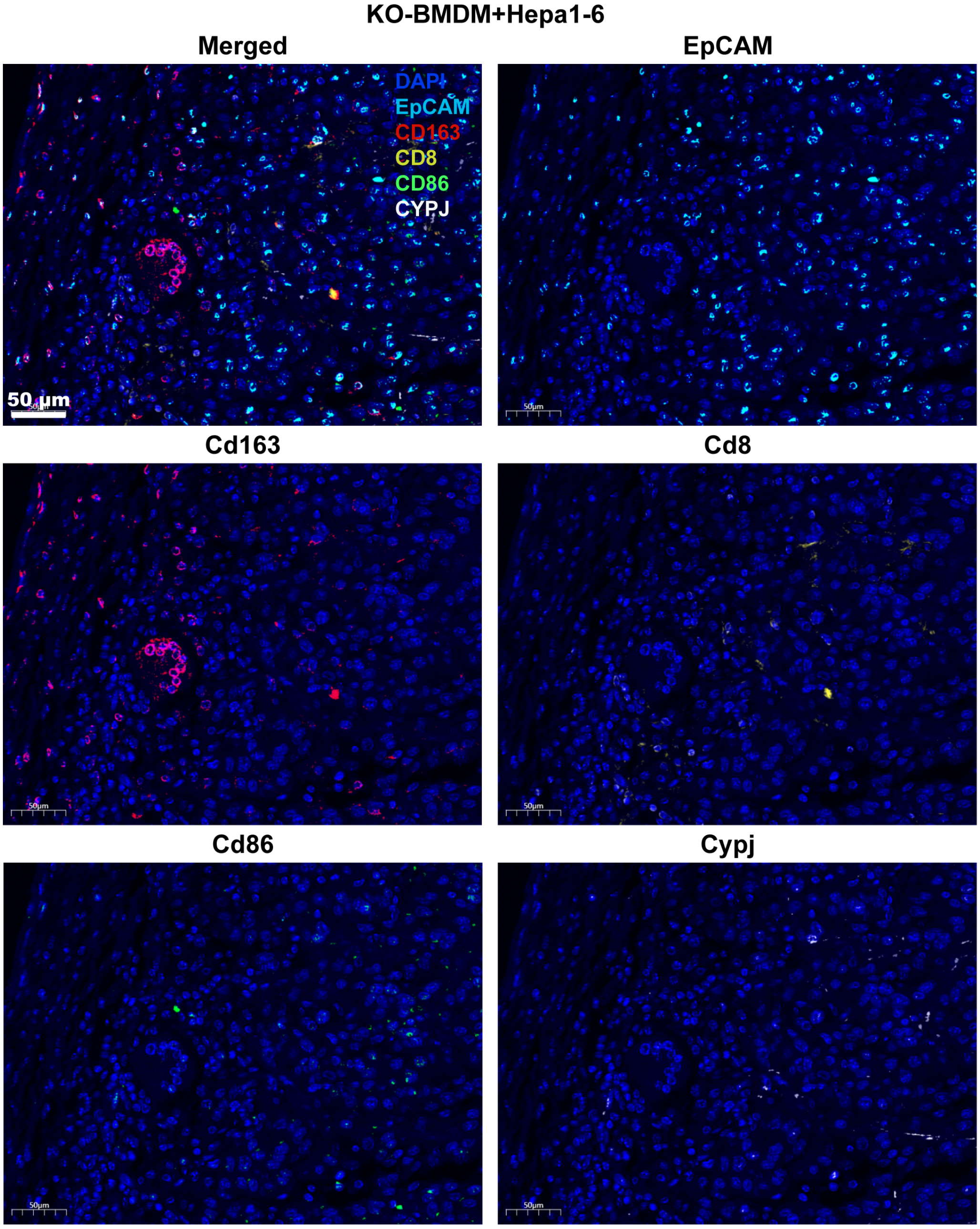

Supplementary Figure 6. Analysis of mIHC in Hepa1-6 Tumors Mixed with KO-BMDM.

mIHC of Hepa1-6 tumors mixed with KO-BMDM labeled with DAPI (blue), CYPJ (white), CD86 (green), CD163 (red), CD8 (yellow), and EpCAM (cyan). Scale bar, 50 μm.

Supplementary Figure 7

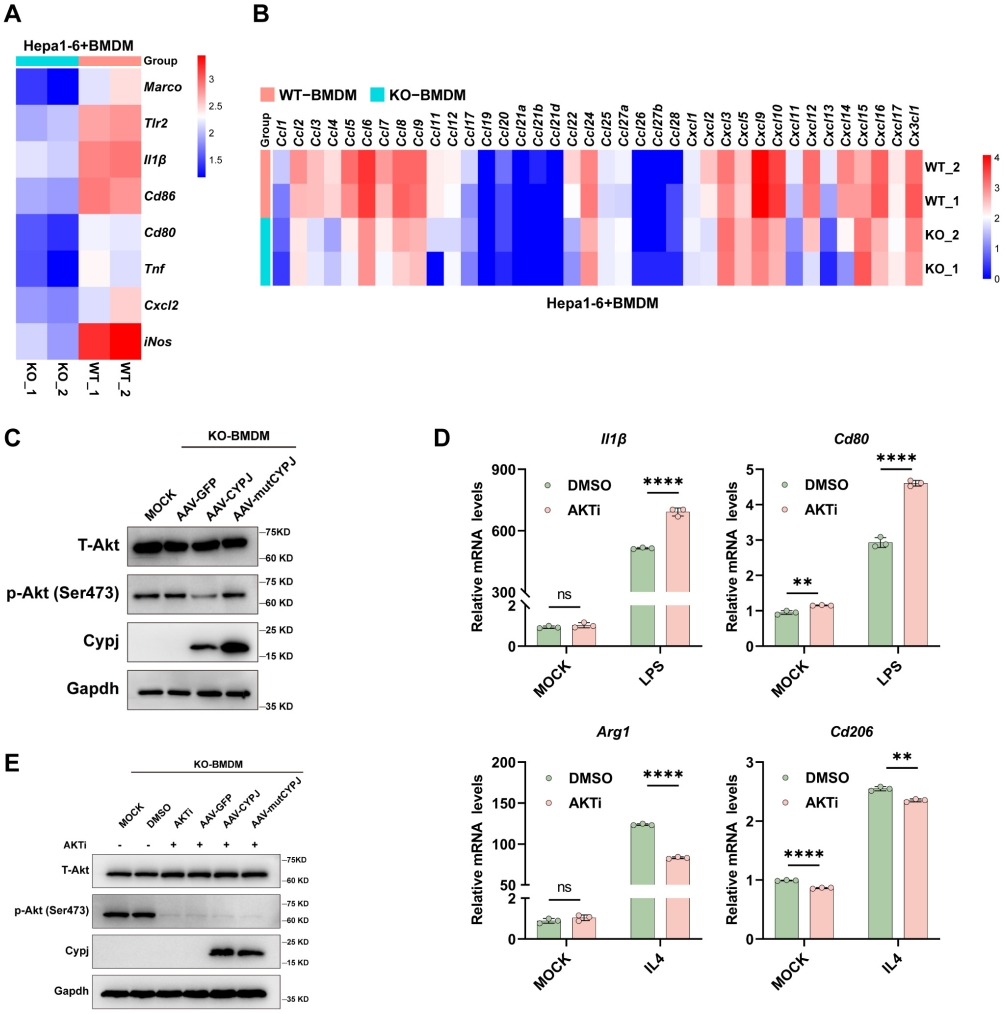

Supplementary Figure 7. Mechanisms of CYPJ Regulates Macrophages.

(**A, B**) Heatmap of markers associated with M1 polarization (**A**) and chemokines (**B**) in tumors of Hepa1-6 cells mixed with WT-BMDM (WT_1 and WT_2) or KO-BMDM (KO_1 and KO_2), with expression levels normalized to $\log_{10}$(count+1). (**C**) WB analysis of AKT in KO-BMDM after AAV infection. (**D**) qRT-PCR detects M1 markers (*Il1β* and *Cd80*) and M2 markers(*Arg1* and *Cd206*) in KO-BMDM after treatment with AKT inhibitor (AKTi) and rAAV virus. **(E)** WB analysis of AKT treated with AKT inhibitor (AKTi). (*p <0.05, ** p <0.01, *** p <0.001, **** p <0.0001, ns=non-significant).

Supplementary Figure 8

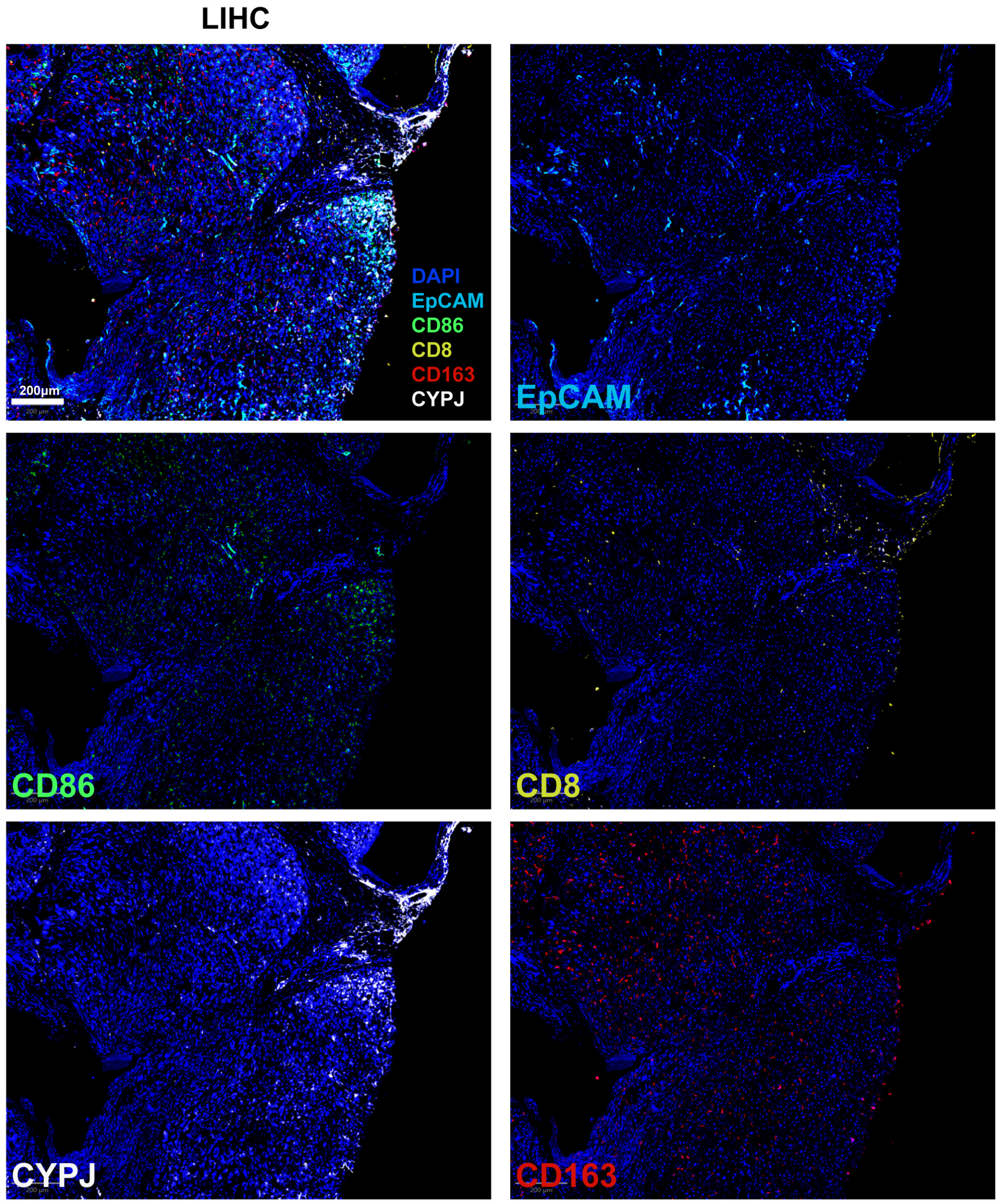

Supplementary Figure 8. Analysis of mIHC in LIHC Patients.

mIHC of liver cancer tissue, labeled with DAPI (blue), CYPJ (white), CD86 (green), CD163 (red), CD8 (yellow), and EpCAM (cyan), then scanned using the Vectra Polaris Pathology Imaging System. Scale bar, 200 μm.

Supplementary Figure 9

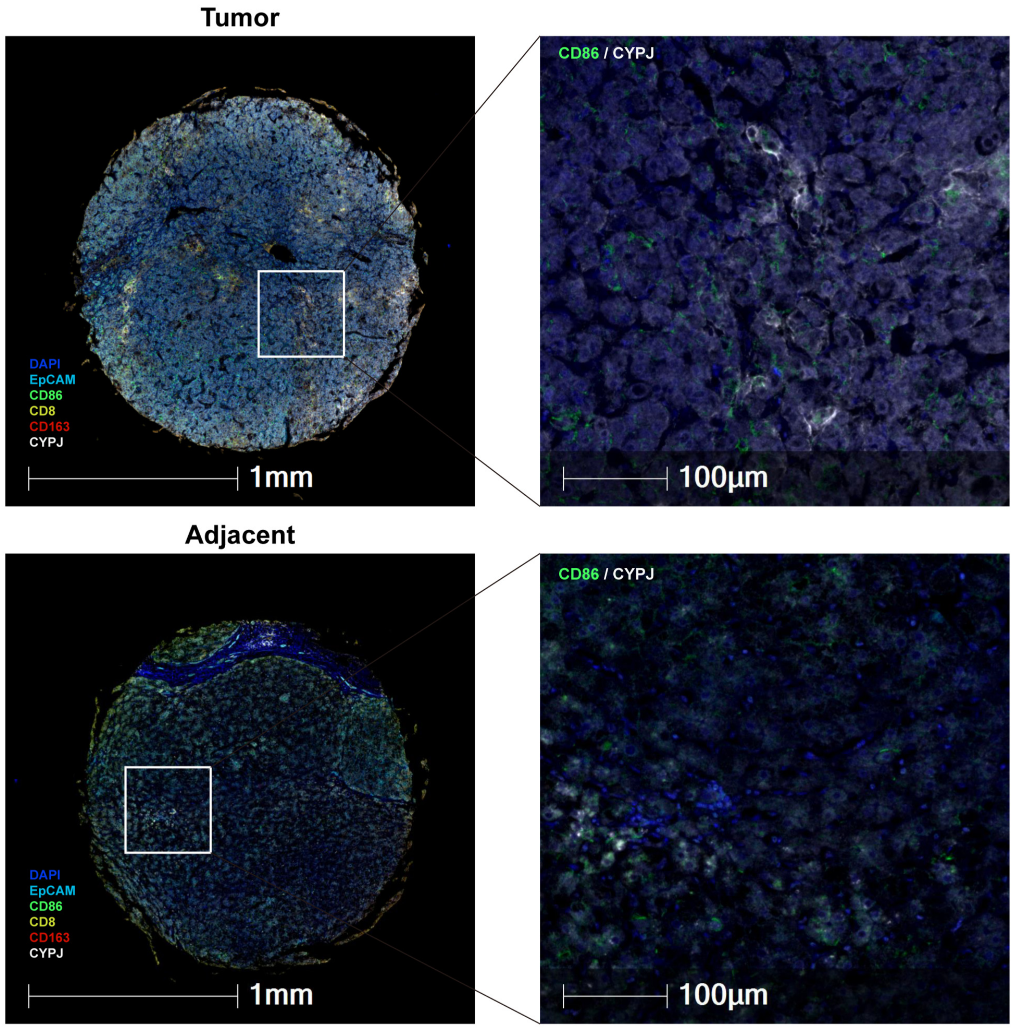

Supplementary Figure 9. Analysis of mIHC in Liver Cancer and Adjacent Normal Tissues.

The mIHC detects the expression of CYPJ, CD86 (M1 TAMs), CD163 (M2 TAMs), CD8 ($CD8^{+}$ T cell), and EpCAM (tumor cell) in liver cancer and adjacent normal tissues. Scale bar, 1 mm and 100 µm.

Supplementary Tables

Supplementary Table 1. Primer sequences of qRT-PCR

Genes	Forward (5'-3')	Reverse (5'-3')
**Mouse**		
IL1β	TGGACCTTCCAGGATGAGGACA	GTTCATCTCGGAGCCTGTAGTG
Cd80	CCTCAAGTTTCCATGTCCAAGGC	GAGGAGAGTTGTAACGGCAAGG
Cd86	ACGTATTGGAAGGAGATTACAGCT	TCTGTCAGCGTTACTATCCCGC
iNos	CTATGGCCGCTTTGATGTGC	TTGGGATGCTCCATGGTCAC
Il10	CGGGAAGACAATAACTGCACCC	CGGTTAGCAGTATGTTGTCCAGC
Arg1	GTAGACCCTGGGGAACACTAT	ATCACCTTGCCAATCCCCAG
Cd206	TCATTCCCTCAGCAAGCGAT	GTGGATACTTGCCAGGTCCC
Cypj	CTGTGAGAGAACACCCAAAACA	TTTTTGGCCCAGATGCTGCTA
Gapdh	CATCACTGCCACCCAGAAGACTG	ATGCCAGTGAGCTTCCCGTTCAG
**Human**		
h*CYPJ*	TGTAGGTGATATTAAAATTGAAGTCTTC	CTTCTTGCCCCAAATACTGTTG

Supplementary Table 2. The information on antibodies

Antibodies	Source	Identifier	Dilution Ratio
**Antibodies for IHC, mIHC, WB and IP**			
Rabbit polyclonal anti-CD86	Proteintech	Cat#26903-1-AP	IHC (1:100)<br>mIHC (1:100)
Mouse monoclonal anti-CD163	OriGene	Cat#TA506381	IHC (1:100)<br>mIHC (1:100)
Mouse monoclonal anti-CD8	Servicebio	Cat#GB12068	mIHC (1:100)
Rabbit polyclonal anti-CYPJ	Abcam	Cat#ab251870	IHC (1:100)<br>mIHC (1:100)<br>WB (1:1000)
Rabbit polyclonal anti-EpCAM	Proteintech	Cat#21050-1-AP	mIHC (1:200)
Rabbit polyclonal anti-GAPDH	HUABIO	Cat#R1210-1	WB (1:5000)
Rabbit polyclonal anti-β Tubulin	Fdbio	Cat#FD0064-100	WB (1:1000)
Rabbit polyclonal anti-mTOR	Cell Signaling Technology	Cat#2983	WB (1:1000)
Rabbit polyclonal anti- PI3K (P85)	Signalway Antibody	Cat#48184	WB (1:1000)
Mouse polyclonal anti-AKT (pan)	Cell Signaling Technology	Cat#2920	WB (1:1000)
Rabbit polyclonal anti- Phospho-AKT (Ser473)	Cell Signaling Technology	Cat#4060	WB (1:1000)

Mouse anti-Myc	Proteintech	Cat#16286-1-AP	WB (1:1000)<br>IP (1 μg /mL)
Mouse anti-Flag	Sigma-Aldrich	Cat#F1804	WB (1:1000)<br>IP (1 μg/mL)
Mouse anti-GFP	Proteintech	Cat#66002-1-AP	WB (1:1000)<br>IP (1 μg/mL)
Mouse IgG	Santa Cruz	Cat#sc-2025	IP (1 μg/mL)
**Antibodies for Flow Cytometry**			
FITC anti-mouse CD45	Biolegend	Cat#103108	1:200
PE anti-mouse CD11b	Biolegend	Cat#101207	1:200
APC anti-mouse CD11b	sungene	Cat#M10117-11A	1:200
APC anti-mouse F4/80	Biolegend	Cat#123116	1:200
BUV737 anti-mouse iNOS	Invitrogen	Cat#367-5920-82	1:100
PE-cy7 anti-mouse CD206	Biolegend	Cat#141719	1:100
PE anti-mouse CD3	Biolegend	Cat#100206	1:200
APC anti-mouse CD4	Elabscience	Cat#E-AB-1097E	1:200
PE-cy7 anti-mouse CD8	Biolegend	Cat#100722	1:200

Supplementary Table 3. Expression of markers associated with M1 polarization

Original count value				
**Group**	Hepa1-6+KO-BMDM	Hepa1-6+KO-BMDM	Hepa1-6+WT-BMDM	Hepa1-6+WT-BMDM
**Gene**	KO_1	KO_2	WT_1	WT_2
Marco	27	14	153	284
Tlr2	87	111	505	579
Il1β	149	125	674	805
Cd86	84	73	768	577
Cd80	36	24	173	161
Tnf	34	14	223	146
Cxcl2	74	59	147	333
Nos2	128	71	1636	2749
**Normalized to log10(count+1)**				
**Group**	Hepa1-6+KO-BMDM	Hepa1-6+KO-BMDM	Hepa1-6+WT-BMDM	Hepa1-6+WT-BMDM
**Gene**	KO_1	KO_2	WT_1	WT_2
Marco	1.447158031	1.176091259	2.187520721	2.45484486
Tlr2	1.944482672	2.049218023	2.704150517	2.763427994
Il1β	2.176091259	2.100370545	2.829303773	2.906335042
Cd86	1.929418926	1.86923172	2.88592634	2.761927838
Cd80	1.568201724	1.397940009	2.240549248	2.209515015
Tnf	1.544068044	1.176091259	2.350248018	2.167317335
Cxcl2	1.875061263	1.77815125	2.170261715	2.523746467
Nos2	2.11058971	1.857332496	3.214048679	3.439332694

Supplementary Table 4. Expression of chemokines

Original count value				
**Group**	Hepa1-6+KO-BMDM	Hepa1-6+KO-BMDM	Hepa1-6+WT-BMDM	Hepa1-6+WT-BMDM
**Gene**	KO_1	KO_2	WT_1	WT_2
Ccl1	2	5	9	38
Ccl2	191	171	398	838
Ccl3	70	57	370	345
Ccl4	36	20	241	210
Ccl5	89	145	888	1528
Ccl6	814	822	3938	4388
Ccl7	90	153	348	689
Ccl8	255	517	1883	1818
Ccl9	343	417	1515	1707
Ccl11	0	52	148	153
Ccl12	90	53	100	142
Ccl17	10	20	14	50
Ccl19	0	0	0	0
Ccl20	1	3	3	7
Ccl21a	0	0	0	0
Ccl21b	0	0	0	1
Ccl21d	0	0	0	0
Ccl22	13	25	108	243
Ccl24	828	486	1222	1039
Ccl25	76	79	55	68
Ccl27a	93	108	50	82
Ccl26	1	0	0	0
Ccl27b	1	0	0	0
Ccl28	1	3	3	0
Cxcl1	62	85	74	79
Cxcl2	74	59	147	333
Cxcl3	1312	1431	897	1310
Cxcl5	343	344	251	290
Cxcl9	998	1179	11987	10800
Cxcl10	438	487	4023	3232
Cxcl11	7	12	34	74
Cxcl12	57	323	1327	1666
Cxcl13	1	4	20	71
Cxcl14	13	128	694	970
Cxcl15	2322	1293	697	479
Cxcl16	350	420	2340	1833
Cxcl17	146	106	132	172
Cx3cl1	740	1021	2403	1563

Normalized to log10(count+1)				
**Group**	Hepa1-6+KO-BMDM	Hepa1-6+KO-BMDM	Hepa1-6+WT-BMDM	Hepa1-6+WT-BMDM
**Gene**	KO_1	KO_2	WT_1	WT_2
Ccl1	0.477121255	0.77815125	1	1.591064607
Ccl2	2.283301229	2.235528447	2.600972896	2.923761961
Ccl3	1.851258349	1.763427994	2.56937391	2.539076099
Ccl4	1.568201724	1.322219295	2.383815366	2.324282455
Ccl5	1.954242509	2.164352856	2.948901761	3.184407485
Ccl6	2.911157609	2.915399835	3.595385981	3.642365581
Ccl7	1.959041392	2.187520721	2.542825427	2.838849091
Ccl8	2.408239965	2.71432976	3.275080898	3.259832699
Ccl9	2.536558443	2.621176282	3.180699201	3.232487866
Ccl11	0	1.72427587	2.173186268	2.187520721
Ccl12	1.959041392	1.73239376	2.004321374	2.155336037
Ccl17	1.041392685	1.322219295	1.176091259	1.707570176
Ccl19	0	0	0	0
Ccl20	0.301029996	0.602059991	0.602059991	0.903089987
Ccl21a	0	0	0	0
Ccl21b	0	0	0	0.301029996
Ccl21d	0	0	0	0
Ccl22	1.146128036	1.414973348	2.037426498	2.387389826
Ccl24	2.918554531	2.687528961	3.087426457	3.017033339
Ccl25	1.886490725	1.903089987	1.748188027	1.838849091
Ccl27a	1.973127854	2.037426498	1.707570176	1.919078092
Ccl26	0.301029996	0	0	0
Ccl27b	0.301029996	0	0	0
Ccl28	0.301029996	0.602059991	0.602059991	0
Cxcl1	1.799340549	1.934498451	1.875061263	1.903089987
Cxcl2	1.875061263	1.77815125	2.170261715	2.523746467
Cxcl3	3.118264726	3.155943018	2.953276337	3.117602692
Cxcl5	2.536558443	2.537819095	2.401400541	2.463892989
Cxcl9	2.999565488	3.071882007	4.078746734	4.033463966
Cxcl10	2.64246452	2.688419822	3.604657972	3.509605705
Cxcl11	0.903089987	1.113943352	1.544068044	1.875061263
Cxcl12	1.763427994	2.51054501	3.123198075	3.2219356
Cxcl13	0.301029996	0.698970004	1.322219295	1.857332496
Cxcl14	1.146128036	2.11058971	2.841984805	2.98721923
Cxcl15	3.36604921	3.111934276	2.843855423	2.681241237
Cxcl16	2.545307116	2.624282096	3.369401414	3.263399331
Cxcl17	2.167317335	2.029383778	2.123851641	2.238046103
Cx3cl1	2.869818208	3.009450896	3.380934463	3.194236749
